# *Hypsizygus marmoreus* polysaccharides protect against cisplatin-induced intestinal mucositis via modulation of gut microbiota, inflammation, and intestinal barrier function

**DOI:** 10.3389/fnut.2026.1774118

**Published:** 2026-04-10

**Authors:** Mohammed Abusidu, Yamina Alioui, Lara Al-tibi, Dhafer Alwayli, Sharafat Ali, Mujeeb Ur Rahman, Nabeel Ahmed Farooqui, Aamna Atta, Bin Feng, Shuming Lu, Liang Wang

**Affiliations:** 1 Department of Biotechnology, College of Basic Medical Science, Dalian Medical University, Dalian, Liaoning Province, China; 2 The Marine Biomedical Research Institute of Guangdong Zhanjiang, School of Ocean and Tropical Medicine, Guangdong Medical University, Zhanjiang, Guangdong, China; 3 Department of Laboratory Medicine Sciences, College of Medicine Faculty of Medical Sciences, Al-Aqsa University, Gaza, Palestine; 4 Department of Pathogen Biology and Microecology, College of Basic Medical Sciences, Dalian Medical University, Dalian, Liaoning Province, China; 5 Department of Biochemistry and Molecular Biology, College of Basic Medical Science, Dalian Medical University, Dalian, Liaoning Province, China; 6 Department of Gastroenterology, First Affiliated Hospital of Dalian Medical University, Dalian, Liaoning Province, China; 7 Stem Cell Clinical Research Center, National Joint Engineering Laboratory, Regenerative Medicine Center, The First Affiliated Hospital of Dalian Medical University, Dalian, Liaoning Province, China

**Keywords:** 16S rRNA sequencing, cisplatin, gut microbiota dysbiosis, *Hypsizygus marmoreus*, intestinal mucositis, polysaccharides, systemic inflammation

## Abstract

**Introduction:**

Chemotherapy-induced intestinal mucositis is a frequent and debilitating complication of anticancer treatment, particularly with cisplatin, leading to disruption of the epithelial barrier, inflammation, and dysbiosis of the gut microbiota. Natural polysaccharides with antioxidant and immunomodulatory properties have attracted increasing attention as potential protective agents. This study investigated the protective effects of *Hypsizygus marmoreus* polysaccharides (HMP) against cisplatin-induced intestinal mucositis in mice.

**Methods:**

HMP were extracted and structurally characterized through monosaccharide composition and molecular weight analysis. Antioxidant activity was evaluated using the ABTS^+^ radical-scavenging assay. A cisplatin-induced mucositis mouse model was established to assess the protective effects of HMP on clinical symptoms, intestinal histopathology, inflammatory cytokines, epithelial barrier integrity, and gut microbiota composition using 16S rRNA sequencing.

**Results:**

HMP were obtained at a yield of 16% and consisted mainly of glucose (52.44%) and galactose (21.05%), together with other monosaccharides, with a molecular weight of 752.96 kDa. *In vitro*, HMP exhibited concentration-dependent antioxidant activity, with ABTS^+^ radical-scavenging increasing from 36.2% at 0.25 mg/mL to nearly 100% at≥20 mg/mL. *In vivo*, cisplatin administration induced body weight loss, diarrhea, immune organ atrophy, intestinal epithelial damage, and gut microbiota dysbiosis. HMP treatment significantly alleviated these alterations, improved intestinal histological structure, enhanced tight junction protein expression and mucin secretion, suppressed pro-inflammatory cytokines including IL-1β, IL-6, and TNF-*α*, and restored microbial diversity. Notably, beneficial taxa such as as *Akkermansia muciniphila* and *Bifidobacterium pseudolongum* were enriched following HMP administration.

**Discussion:**

These findings indicate that HMP protects against cisplatin-induced intestinal mucositis through multiple complementary mechanisms, including antioxidant activity, suppression of inflammatory responses, preservation of epithelial barrier integrity, and modulation of gut microbiota composition. HMP therefore represents a promising natural candidate for mitigating chemotherapy-induced gastrointestinal toxicity.

## Graphical abstract

**Figure:**
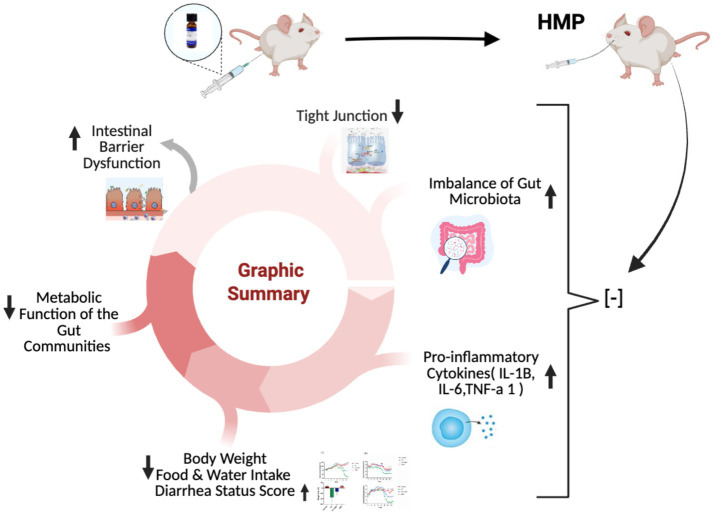
*Hypsizygus marmoreus* polysaccharides (HMP) protect against cisplatin-induced intestinal toxicity by preserving epithelial barrier integrity, reducing inflammation and modulating gut microbiota. In mice, cisplatin induces weight loss, diarrhea, immune organ atrophy, mucosal inflammation, tight junction disruption, and goblet cell depletion. Oral HMP mitigates these effects by restoring junction proteins (claudin-1, occludin, ZO-1), increasing Mucin-2, reducing systemic and ileal proinflammatory cytokines, improving clinical and histological scores, and partially normalizing microbial diversity and community structure.

## 1 Introduction

Mucositis is a common and debilitating complication of radiotherapy, chemotherapy, and combined chemoradiotherapy (CRT), affecting the gastrointestinal tract from the oral cavity to the rectum (1). Clinically, it manifests as painful ulcerations accompanied by anorexia, nausea, and diarrhea, often necessitating opioid analgesia and leading to treatment delays or discontinuation (2). Despite advances in supportive care, mucositis remains highly prevalent, affecting up to 80% of patients receiving cytotoxic chemotherapy or radiation-based therapies and approaching universal incidence in those receiving high-dose chemotherapy (3, 4).

Cisplatin (CP), a platinum-based chemotherapeutic agent, remains highly effective against various solid tumors, including lung, breast, bladder, and ovarian cancers. However, its non-selective cytotoxicity causes severe side effects such as nephrotoxicity, neurotoxicity, myelosuppression, and gastrointestinal (GI) mucositis (5). Up to 40–100% of patients experience GI injury during chemotherapy, presenting with diarrhea, abdominal pain, and malnutrition (6, 7). The pathogenesis of CP-induced mucositis follows Sonis’ five-stage model, initiated by DNA damage and excessive reactive oxygen species (ROS) generation, followed by NF-κB activation, cytokine release, epithelial apoptosis, bacterial translocation, and systemic inflammation (8). This cascade leads to barrier dysfunction and microbial imbalance, thereby aggravating mucosal inflammation.

Despite its clinical and economic burden, current mucositis therapies are largely palliative. Only palifermin and benzydamine have been approved for clinical use, both with limited efficacy and accessibility. Other strategies, including cryotherapy, photo biomodulation, immunonutrition and cytokine-based treatments, require further validation (9).

Recently, immunonutrition (IN) has emerged as a supportive strategy, providing specific nutrients to modulate inflammatory responses and counteract treatment-induced immune impairment (10). The effects of selected substances, such as omega-3 fatty acids, glutamine, and nucleotides, on the secretion of inflammatory mediators including cytokines, chemokines, and interferons have been demonstrated (11). These immunonutrients can stabilize cellular membranes and inhibit pro-inflammatory pathways (12, 13), potentially reducing the severity of toxicities such as oral mucositis and diarrhea while supporting weight recovery and overall treatment outcomes (14). Nevertheless, no universally effective prophylactic or therapeutic agent currently exists to prevent chemotherapy-induced mucositis.

The intestinal epithelium forms a vital barrier between the host and luminal contents (15). Chemotherapy-induced oxidative stress and inflammation disrupt tight junctions, increasing permeability and promoting bacterial translocation (8, 16). Concurrently, gut microbiota imbalance contributes to mucosal injury by impairing epithelial regeneration and reducing short-chain fatty acid (SCFA) production (17). CP induces marked dysbiosis, characterized by reduced beneficial bacteria (*Lactobacillus*, *Bifidobacterium*) and increased opportunistic pathogens (*Enterococcus*, *Pseudomonas*), further aggravating inflammation (18). Chemotherapy-induced mucositis remains a major clinical challenge, with limited effective adjuvant strategies available for its prevention. Natural bioactive compounds, particularly medicinal mushrooms, have attracted growing interest for their potential to alleviate mucosal injury and enhance treatment tolerance due to their immunoregulatory and antioxidant properties (19). Among their active constituents, polysaccharides act as prebiotic fibers that regulate gut microbiota composition and modulate immune and inflammatory responses (20, 21). *Hypsizygus marmoreus*, an edible and medicinal mushroom widely consumed in Asia, is rich in polysaccharides composed mainly of rhamnose, mannose, galactose, and glucose. These biopolymers exhibit notable antioxidant, immunomodulatory, and microbiota-regulating activities (22, 23). This study investigated the protective effects of *H. marmoreus* polysaccharides (HMP) against cisplatin-induced intestinal mucositis. We hypothesized that HMP could mitigate mucosal injury by restoring microbial balance, suppressing inflammation and oxidative stress, and enhancing epithelial barrier integrity.

## 2 Materials and methods

### 2.1 Chemicals and reagents

Cisplatin (CP) was purchased from Qilu Pharmaceutical Co., Ltd. (Jinan, China). Stool DNA was isolated using a kit obtained from FORGENE (Chengdu, China). ELISA kits for mouse TNF-*α*, IL-1β, and IL-6 were provided by Jonlnbio Co., Ltd. (Shanghai, China). Phosphate-buffered saline (PBS) powder and the immunohistochemistry (IHC) detection kit were supplied by Beijing Zhongshan Jinqiao Biotechnology Co., Ltd. (Beijing, China). Primary antibodies against Claudin-1, Occludin, ZO-1, and Mucin-2, as well as the corresponding secondary antibodies, were purchased from Proteintech Group (Wuhan, China).

### 2.2 *Hypsizygus marmoreus* polysaccharides

Dried fruiting bodies of *Hypsizygus marmoreus* were ground into a fine powder and defatted with 80% ethanol at 60 °C for 2 h. The residue was filtered, air-dried, and then mixed with distilled water at a ratio of 1:20 (w/v). Hot-water extraction was performed at 95 °C for 3 h under continuous stirring. The extract was subsequently subjected to ultrasonic treatment at 67% power for 20 min, and the pH was adjusted to 7.4 using 1 M NaOH. The mixture was centrifuged at 4000 × g for 15 min, and the supernatant was filtered through a 0.45 μm membrane. The filtrate was concentrated to one-fifth of its original volume using a rotary evaporator at 60 °C. Polysaccharides were then precipitated by adding four volumes of absolute ethanol, followed by overnight incubation at 4 °C. The resulting precipitate was collected by centrifugation (4,000 × g, 15 min, 4 °C), frozen at −80 °C, and lyophilized for 48 h to obtain dry *H. marmoreus* polysaccharide (HMP) powder. The extraction yield was calculated as follows (Equation 1):


$$\text{Yield}\;(\%)=\frac{\text{Mushroom weight}\;(g)}{\text{Polysaccharide weight}\;(g)}\times 100$$ (1)

Total carbohydrate content was determined using the phenol-sulfuric acid method, and protein content was quantified by the Bradford assay.

### 2.3 Molecular weight determination

The molecular weight distribution of the polysaccharide extract was determined by size-exclusion chromatography coupled with multi-angle laser light scattering and refractive index detection (SEC-MALLS-RI). The analysis was performed using a DAWN HELEOS-II MALS detector and an Optilab T-rEX RI detector (Wyatt Technology, United States) connected to tandem Shodex OHpak SB-805 and SB-803 columns (8 × 300 mm; Showa Denko, Japan). The mobile phase consisted of 0.1 M NaNO₃ containing 0.02% NaN₃, delivered at a flow rate of 0.6 mL/min. The column temperature was maintained at 45 °C, and the injection volume was 20 μL. Prior to analysis, samples were dissolved in the same solvent at 1 mg/mL and filtered through 0.45 μm membranes. The weight-average (Mw) and number-average (Mn) molecular weights, as well as the polydispersity index (Mw/Mn), were calculated using ASTRA 6.1 software (Wyatt Technology). The refractive index increment (dn/dc) was determined to be 0.141 mL/g under identical solvent conditions.

### 2.4 Monosaccharide composition analysis

Monosaccharide composition of the polysaccharide sample was determined by high-performance anion-exchange chromatography coupled with pulsed amperometric detection (HPAEC-PAD). Approximately 5 mg of HMP was hydrolyzed with 2 M trifluoroacetic acid (TFA) at 121 °C for 2 h in a sealed tube. The hydrolysate was evaporated to dryness under a nitrogen stream, washed repeatedly (2–3 times) with methanol, and re-dissolved in deionized water. The solution was filtered through a 0.22 μm membrane prior to analysis. HPAEC-PAD analysis was performed on a Dionex ICS-5000 + system (Thermo Fisher Scientific, United States) equipped with a CarboPac PA20 column (3 × 150 mm). The mobile phase consisted of solvent A (ddH₂O), solvent B (0.1 M NaOH), and solvent C (0.1 M NaOH + 0.2 M sodium acetate), delivered at a flow rate of 0.5 mL/min. The gradient program was as follows: 0 min, A/B/C = 95:5:0; 26 min, 85:5:10; 42 min, 85:5:10; 42.1 min, 60:0:40; 52 min, 60:40:0; 52.1 min, 95:5:0; and 60 min, 95:5:0. The injection volume was 5 μL. Chromatographic data were acquired and processed using Chromeleon 7.2 CDS software (Thermo Fisher Scientific), and the monosaccharide components were quantified by comparison with standard sugars.

### 2.5 Total antioxidant capacity assay

Total antioxidant capacity (T-AOC) was measured using a commercial ABTS assay kit (ABTS method) according to the manufacturer’s instructions. Reagents A and B were dissolved in PBS, mixed at a 1:1 (v/v) ratio, incubated in the dark at room temperature for 12 h, and diluted 40-fold with PBS to obtain the ABTS^+^ working solution. A Trolox stock solution (4 μmol/mL) was prepared by dissolving 2 mg Trolox in 2 mL ethanol and diluted with PBS to obtain standards ranging from 0 to 0.4 μmol/mL. Polysaccharide extracts (0.25–25 mg/mL) were prepared in PBS. In a 96-well microplate, 10 μL of sample was mixed with 190 μL of ABTS^+^ working solution and incubated at 25 °C in the dark for 6 min. A blank (ABTS^+^ solution without sample) and a control (sample without ABTS^+^ solution) were included. All measurements were performed in triplicate. Absorbance was read at 414 nm, and the corrected absorbance was calculated as follows (Equation 2):


$$\mathrm{\Delta A}=A_{\text{blank}}-(A_{\text{sample}}-A_{\text{control}})$$ (2)

Antioxidant activity was determined using the Trolox standard curve (0–0.4 μmol/mL) and expressed as μmol Trolox equivalents per milliliter (μmol TE/mL) according to the obtained equation. The ABTS radical-scavenging activity (%) was calculated as follows (Equation 3):


$$\text{Scavenging activity}\;(\%)=\frac{A_{\text{blank}}-(A_{\text{sample}}-A_{\text{control}})}{A_{\text{blank}}}\times 100$$ (3)

The IC₅₀ (concentration of extract required to scavenge 50% of ABTS^+^ radicals) was determined from the dose–response curve by linear interpolation of the scavenging activity (%) vs. polysaccharide concentration.

### 2.6 Experimental animal

Ethical approval for the animal procedures in this study was granted by the Animal Ethics Committee of Dalian Medical University (Reference number: 202410368, dated May 24, 2024). All procedures were conducted in accordance with the National Institutes of Health guidelines for the care and use of laboratory animals.

Thirty-two male BALB/c mice (5–6 weeks old, 20 ± 2 g) were obtained from the SPF Animal Facility of Dalian Medical University (Dalian, China). The mice were housed in sterilized cages under controlled environmental conditions (22 ± 2 °C, 65 ± 5% relative humidity, and a 12-h light/dark cycle) with free access to standard chow and distilled water.

### 2.7 Establishment of the cisplatin-induced intestinal mucositis model and treatment protocol

Following a 7-day acclimatization period, 32 male mice were randomly assigned to four experimental groups (eight mice per group): Control (normal control), CP (cisplatin-induced model), CP + HMP (cisplatin with HMP treatment), and HMP (HMP-alone positive control). Intestinal mucositis was induced in the CP and CP + HMP groups by intraperitoneal (IP) injection of cisplatin at a dose of 6 mg/kg/day from day 8 to day 11. HMP treatment was administered to the HMP and CP + HMP groups by oral gavage at a dose of 10 mg/kg dissolved in 0.9% sterile saline (vehicle) once daily from day 1 to day 15. Mice in the Control and CP groups received daily oral gavage of an equivalent volume of the vehicle control, 0.9% sterile saline. The experimental design is outlined in section 3.3.

Following a 7-day acclimatization period, 32 male mice were randomly assigned to four experimental groups (eight mice per group): Control (normal control), CP (cisplatin-induced model), CP + HMP (cisplatin with HMP treatment), and HMP (HMP-alone positive control). Intestinal mucositis was induced in the CP and CP + HMP groups by intraperitoneal (IP) injection of cisplatin at 6 mg/kg/day from day 8 to day 11. This dose was selected based on the manufacturer’s recommended protocol for cisplatin-induced toxicity in mice and supported by previously published studies demonstrating reliable induction of intestinal injury with minimal mortality (24). HMP treatment was administered to the HMP and CP + HMP groups by oral gavage at 10 mg/kg dissolved in 0.9% sterile saline (vehicle) once daily from day 1 to day 15. The HMP dose was determined from a preliminary study evaluating three concentrations (5, 10, and 15 mg/kg), in which 10 mg/kg showed the most consistent improvement in intestinal morphology and inflammatory markers without observable adverse effects. Mice in the Control and CP groups received daily oral gavage of an equivalent volume of vehicle (0.9% sterile saline). The experimental design is outlined in section 3.3.

### 2.8 Mucositis assessment and sample collection

During the model establishment and treatment protocol, critical parameters such as body weight, food and water intake, coat condition, and diarrhea status were daily monitored. Diarrheal severity in the fecal samples was graded using the Bowen index (25), with classifications as follows: Normal Particle (Grade-0), Mildly Soft Stool (Grade-1), Moderately Soft Stool (Grade-2), and Watery Stool or shapeless stool (Grade-3) (26). On the 15th day of the experiment, fecal samples were collected and stored at −80 °C for microbiome analysis. On day 16th of the experiment, mice were humanely euthanized, and various samples, including distal colon, ileum tissues were collected, fixed in 4% formalin for histopathological examination, while blood, and colon tissues were collected and stored at −80 °C for molecular studies.

### 2.9 Measurement of organ indices

The spleen and thymus indices were used to evaluate changes in immune organ development and function. After euthanasia by cervical dislocation, the spleen and thymus were carefully excised and weighed immediately. The organ index (mg/g) was calculated as follows (Equation 4):


$$\text{Organ index}\;(\mathrm{mg}/g)=\frac{\text{Organ weight}\;(\mathrm{mg})}{\text{Body weight}\;(g)}$$ (4)

### 2.10 Histological assessment and evaluation of intestinal barrier integrity

Histological and immunohistochemical analyses were conducted to evaluate intestinal tissue morphology, barrier integrity, and mucin expression. Colon and ileum samples were fixed in 4% paraformaldehyde, dehydrated through graded ethanol, cleared in xylene, embedded in paraffin, and sectioned at 4 μm. Sections were stained with hematoxylin and eosin (H&E) to assess general tissue architecture, epithelial integrity, and inflammatory cell infiltration. Histological scoring was performed based on standardized criteria. For regeneration, scores were defined as follows: 4 = no tissue repair, 3 = surface epithelium not intact, 2 = regeneration with crypt depletion, 1 = almost complete regeneration, and 0 = complete regeneration or normal tissue morphology. For inflammation, 3 = severe, 2 = moderate, 1 = slight, and 0 = none.

For immunohistochemistry (IHC) and immunofluorescence (IF), rehydrated tissue sections underwent antigen retrieval, peroxidase inactivation, and blocking. Sections were incubated with primary antibodies targeting ZO-1 and Mucin-2 for IHC, and Claudin-1 and Occludin for IF, as detailed in Supplementary Table S1. For IHC, slides were incubated with HRP-conjugated secondary antibodies, and signals were developed using DAB, followed by hematoxylin counterstaining. For IF, sections were incubated with appropriate fluorophore-conjugated secondary antibodies, and nuclei were counterstained with DAPI. All stained slides were examined under a light microscope (IHC) and a fluorescence microscope (IF; Leica Microsystems, Wetzlar, Germany).

### 2.11 Determination of systemic pro-inflammatory cytokines

Whole blood samples were collected via retro-orbital puncture, and serum was separated by centrifugation at 3,000 rpm for 10 min at 4 °C. The supernatant was stored at −20 °C until further analysis. Serum levels of interleukin-1β (IL-1β), interleukin-6 (IL-6), and tumor necrosis factor-*α* (TNF-α) were quantified using commercial mouse ELISA kits (Jonlnbio Co., Ltd., Shanghai, China) following the manufacturer’s instructions. Briefly, 100 μL of standards or serum samples were added to the wells and incubated with biotin-labeled antibodies, followed by washing and incubation with streptavidin-horseradish peroxidase (HRP). After a final washing step, tetramethylbenzidine (TMB) substrate solution was added for color development, and the reaction was stopped with a stop solution. The optical density was measured at 450 nm using a microplate reader.

### 2.12 Real-time quantitative PCR

RT-qPCR was performed to quantify the relative mRNA expression of tight junction genes (ZO-1, Occludin, Claudin-1) and the mucin-related gene Mucin-2 in colon tissues. Total RNA was extracted using TRIzol® reagent (Invitrogen, United States) following the manufacturer’s protocol, and further purified by chloroform extraction, isopropanol precipitation, and ethanol washing. RNA concentration and purity were assessed using a NanoDrop 2000 spectrophotometer (Thermo Fisher Scientific, United States). After removal of genomic DNA, 2 μg of total RNA was reverse-transcribed into complementary DNA (cDNA) using the HiScript II Q RT SuperMix kit (Vazyme, China). Quantitative PCR was conducted with ChamQ SYBR qPCR Master Mix (Vazyme, China) on a Bioer LightGene 9,600 system. Relative gene expression levels were calculated using the 2^−ΔΔCt method, with GAPDH as the internal reference. Primer sequences are listed in Supplementary Table S2.

### 2.13 Gut microbiota 16S rRNA gene sequencing

Total genomic DNA was extracted from freshly collected fecal samples using the PowerMax® Soil DNA Isolation Kit (Qiagen, United States) according to the manufacturer’s protocol. Extracted DNA was stored at −80 °C until further analysis. DNA concentration and purity were determined using a NanoDrop spectrophotometer (Thermo Fisher Scientific, Waltham, MA, USA), and integrity was verified by agarose gel electrophoresis. The V4 hypervariable region of the bacterial 16S rRNA gene was amplified using universal primers 515F (5′-GTGCCAGCMGCCGCGGTAA-3′) and 806R (5′-GGACTACHVGGGTWTCTAAT-3′). PCR amplification was conducted under the following conditions: initial denaturation at 98 °C for 30 s, followed by 25 cycles of denaturation at 98 °C for 15 s, annealing at 58 °C for 15 s, and extension at 72 °C for 15 s, with a final extension at 72 °C for 1 min. Amplicons were purified, quantified, and sequenced on the Illumina NovaSeq 6,000 platform (Illumina, San Diego, CA, United States). Raw reads were subjected to quality control to remove chimeric and low-quality sequences using QIIME (version 1.9.1). Operational taxonomic units (OTUs) were clustered at 97% sequence similarity, and taxonomic classification was performed using the Greengenes reference database (version 13_8). Alpha diversity indices, including Chao1 richness, Shannon, and Simpson evenness, were calculated using QIIME and R software (version 3.2.0). Beta diversity was evaluated based on UniFrac distance metrics and visualized using principal coordinate analysis (PCoA), principal component analysis (PCA), and nonmetric multidimensional scaling (NMDS).

### 2.14 Bioinformatics and metagenomic functional analysis

The functional composition of the gut microbiome was analyzed computationally. 16S rRNA data were used to predict microbial functions using PICRUSt. KEGG pathways were used to link genetic and metabolic information. Further analysis was conducted with STAMP, FAPROTAX and BugBase.

### 2.15 Statistical analysis

Statistical data were analyzed using GraphPad Prism (version 10.01). One-way analysis of variance (ANOVA) and Tukey’s multiple comparison tests were employed. A *p*-value < 0.05 was considered statistically significant. For OTU and phenotype data, nonparametric tests (Kruskal-Wallis, Wilcoxon, Mann–Whitney) were used.

## 3 Results

### 3.1 Structural characterization of *Hypsizygus marmoreus* polysaccharides

**Figure 1:**
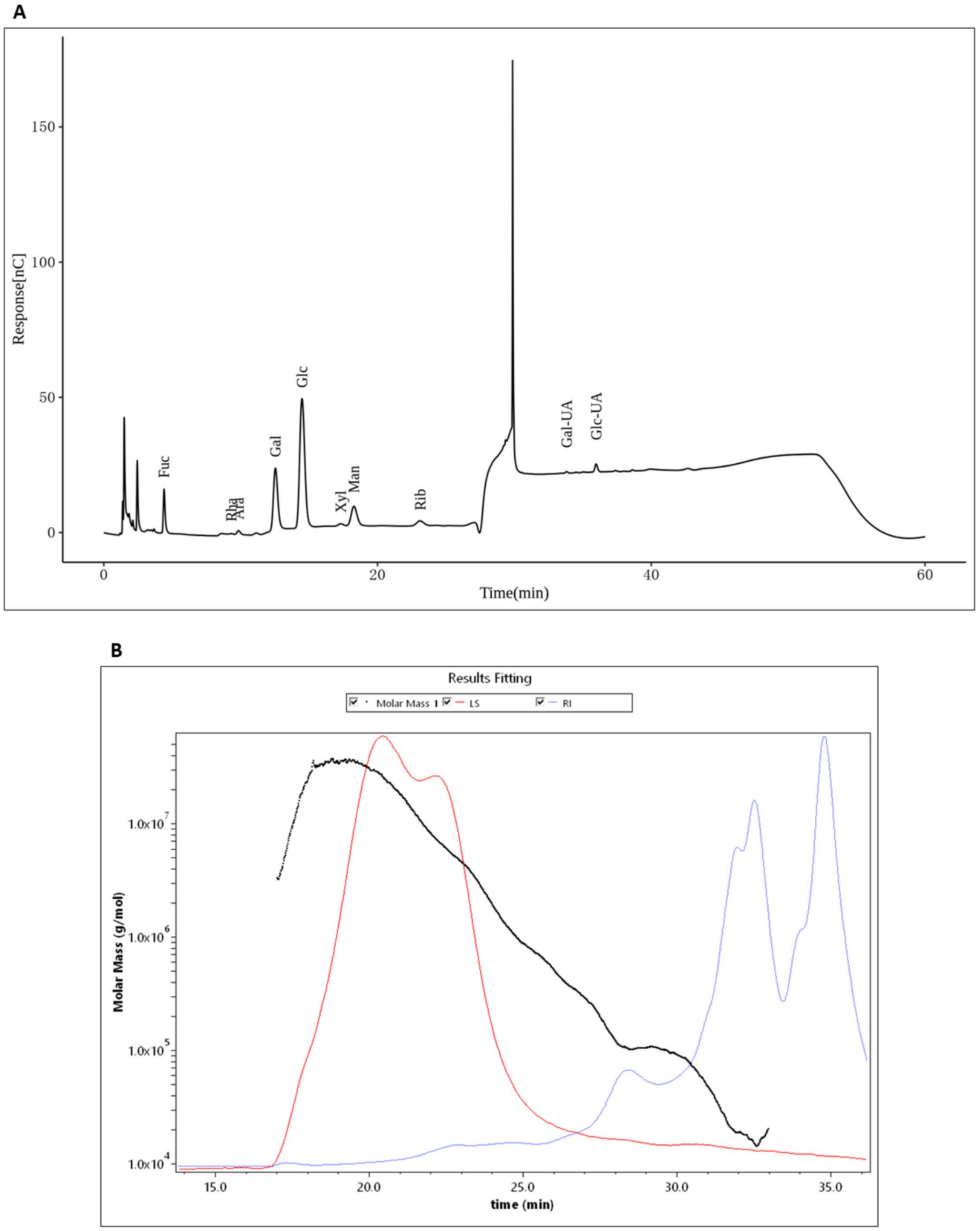
Carbohydrate composition and molar-mass distribution of HMP. **(A)** High-performance anion exchange chromatography with pulsed amperometric detection (HPAEC-PAD) showing neutral sugars and uronic acids identified by retention time: Fuc, Rha, Ara, Gal, Glc, Xyl, Man, Rib, GalA, and GlcA. Signal is plotted as detector response vs. elution time (min). **(B)** Size exclusion chromatography coupled with multi angle light scattering and refractive index detection (SEC-MALS/RI). The black trace shows the fitted molar mass (g/mol) over elution time; red and blue traces correspond to light-scattering (LS) and RI signals, respectively, indicating a broad, polydisperse distribution with high-molar-mass species eluting earlier.

HMP were obtained as a crude extract with an extraction yield of 16%. The total carbohydrate concentration reached 26 mg/mL, with a residual protein content of 2.3%. The monosaccharide composition of HMP was analyzed using high-performance anion-exchange chromatography coupled with pulsed amperometric detection (HPAEC-PAD). The results indicated that HMP is mainly composed of glucose (52.44%), followed by galactose (21.05%) and mannose (9.08%), while fucose (8.55%), ribose (3.11%), and glucuronic acid (2.92%) were present in smaller proportions (Figure 1A; Table 1). This composition identifies HMP as a glucose-rich heteropolysaccharide containing both neutral and acidic sugar residues. Molecular weight analysis revealed a weight-average molecular weight (Mw) of 752.96 kDa and a number-average molecular weight (Mn) of 32.43 kDa, resulting in a polydispersity index (Mw/Mn) of 23.22, indicative of a broad molecular weight distribution. The hydrodynamic radii (Rn, Rw, Rz) were 24.07, 30.16, and 35.75 nm, respectively (Figure 1B; Table 2). Overall, these results demonstrate that crude HMP consist mainly of glucose- and galactose-rich high-molecular-weight polysaccharides, exhibiting structural heterogeneity characteristic of mushroom-derived bioactive polymers.

**Table 1:** Monosaccharide composition and basic chemical characteristics of polysaccharides extracted from *Hypsizygus marmoreus* (HMP).

Monosaccharide composition		
Component	Concentration (ug/mg)	Percentage (%)
Fuc	22.6149	8.55%
Ara	2.5609	0.97%
Rha	0.498	0.19%
Gal	55.6467	21.05%
Glc	138.6575	52.44%
Xyl	2.5185	0.95%
Man	24.0184	9.08%
Rib	8.231	3.11%
Gal-UA	1.9185	0.73%
Glc-UA	7.726	2.92%
Total	264.3904	100%
Total carbohydrates (mg/mL)	26	
Protein content (%)	2.3	

**Table 2:** Molecular weight distribution and polydispersity of *Hypsizygus marmoreus* polysaccharides (HMP).

Molecular weight	
Parameter	Value
Mn (kDa)	32.428
Mp (kDa)	15.576
Mw (kDa)	752.963
Mz (kDa)	16626.617
Polydispersity (Mw/Mn)	23.22
Rn (nm)	24.065
Rw (nm)	30.159
Rz (nm)	35.749
Mass fraction (%)	100

### 3.2 Total antioxidant capacity of HMP

The antioxidant activity of HMP was evaluated using the ABTS^+^ radical-scavenging assay. As shown in Table 3, HMP exhibited a clear concentration-dependent increase in total antioxidant capacity (T-AOC). T-AOC values increased from 0.0495 μmol TE/mL at 0.25 mg/mL to 0.1153 μmol TE/mL at 25 mg/mL.

**Table 3:** Total antioxidant capacity (T-AOC) and ABTS^+^ radical-scavenging activity of *H. marmoreus* polysaccharide extract (HMP) at different concentrations.

Concentration (mg/mL)	T-AOC (μmol TE/mL)	Scavenging (%)
0.25	0.0495 ± 0.00325	36.22 ± 3.17
0.5	0.0515 ± 0.00322	38.23 ± 3.15
1	0.0552 ± 0.00326	41.79 ± 3.19
5	0.0893 ± 0.00326	75.11 ± 3.19
10	0.1126 ± 0.00467	97.88 ± 4.57
15	0.1121 ± 0.00332	97.44 ± 3.24
20	0.1148 ± 0.00338	100 ± 3.30
25	0.1153 ± 0.00341	100.50 ± 3.33

Values are expressed as mean ± SD (*n* = 3). *Values slightly above 100% are due to experimental variability as the radical-scavenging activity approaches a plateau.

Consistently, the ABTS^+^ radical-scavenging rate increased from 36.22% at 0.25 mg/mL to nearly complete inhibition (100%) at concentrations ≥ 20 mg/mL. A marked enhancement in antioxidant activity was observed between 1 and 10 mg/mL, where scavenging activity increased from 41.79 to 97.88%, indicating a strong dose–response relationship. At higher concentrations (15–25 mg/mL), the scavenging activity plateaued, suggesting saturation of radical-neutralizing capacity.

The half-maximal inhibitory concentration (IC₅₀), calculated from the dose–response curve, was 2.0 ± 0.2 mg/mL, indicating moderate antioxidant potency of HMP *in vitro*.

These findings demonstrate that HMP possesses significant concentration-dependent free radical-scavenging activity, supporting its potential role in mitigating oxidative stress associated with cisplatin-induced intestinal injury.

### 3.3 HMP attenuated cisplatin-induced body weight loss and improved general condition

**Figure 2:**
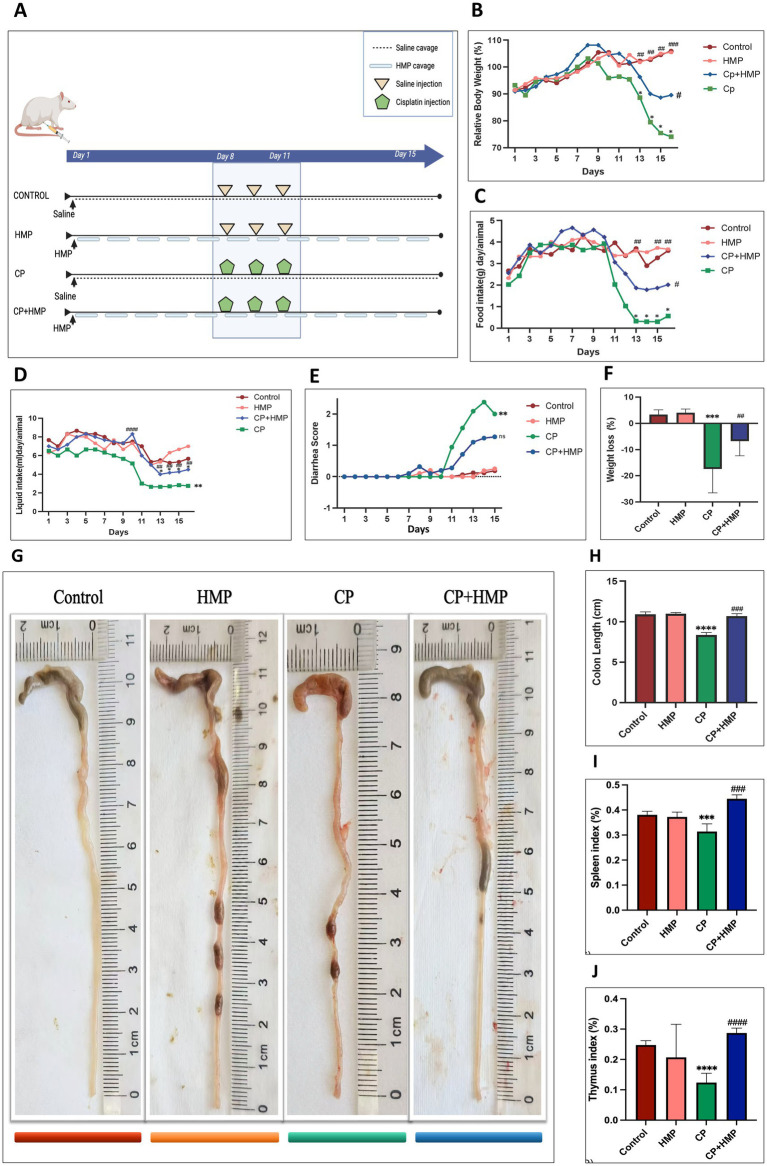
HMP mitigates cisplatin-induced gastrointestinal toxicity and systemic atrophy in mice. **(A)** Experimental design. Mice received daily saline or HMP by gavage, and cisplatin (CP) or saline was administered by injection on days 8–11. **(B–D)** Longitudinal measurements of body weight **(B)**, food intake **(C)**, and water intake **(D)**. CP induced progressive reductions in all parameters, whereas HMP co-treatment (CP+HMP) partially restored these effects. **(E)** Diarrhea scores over time. CP induced severe diarrhea from day 11 onward, while HMP delayed onset and reduced severity. **(F)** Net body weight loss at the experimental endpoint. HMP attenuated CP-induced weight loss. **(G)** Representative images of the colon. CP treatment resulted in shortened and darkened colons with visible lesions, whereas HMP preserved colonic morphology. **(H)** Quantification of colon length. CP significantly shortened colon length, whereas HMP significantly ameliorated this effect. **(I, J)** Thymus and spleen indices. CP significantly reduced both indices, while HMP co-treatment partially restored them. Data are presented as mean ± SD. Statistical significance is indicated relative to the control and CP groups.

Cisplatin administration caused marked systemic toxicity, as evidenced by pronounced weight loss, reduced food and water intake, and severe diarrhea (Figures 2B–F). Throughout the experimental period, mice in the control and HMP-alone groups maintained stable body weight, normal feeding behavior, and no signs of diarrhea, confirming that HMP administration alone was well tolerated and physiologically safe. In contrast, mice in the cisplatin (CP) group displayed a rapid and progressive decline in body weight starting around day 10, reaching an average 18% loss by the end of treatment (*p* < 0.001 vs. Control). Food and water consumption were significantly reduced during the final phase, reflecting cisplatin-induced anorexia and dehydration. Moreover, diarrhea scores in the CP group rose sharply from day 11 onward, peaking at the late stage of the experiment (mean score 3.5 ± 0.3), indicative of severe intestinal mucositis and fluid imbalance.

Notably, HMP supplementation markedly alleviated these systemic effects. Mice in the CP + HMP group exhibited only a moderate 10.5% weight loss (*p* < 0.01 vs. CP), and their food and liquid intake remained significantly higher than that of the CP group. The severity and onset of diarrhea were substantially reduced, with mean scores remaining below 2.0 throughout the treatment course. These results indicate that HMP effectively mitigates cisplatin-induced cachexia, anorexia, dehydration, and diarrhea, underscoring its potential to enhance treatment tolerance and counteract chemotherapy-related gastrointestinal toxicity.

### 3.4 HMP alleviated colonic shortening and tissue damage

As illustrated in Figures 2H,I, mice receiving HMP alone displayed no morphological abnormalities, maintaining colon dimensions comparable to controls. In contrast, cisplatin treatment led to a marked reduction in colon length (*p* < 0.0001) compared with the control group, reflecting the severity of intestinal injury. When HMP was co-administered with cisplatin, the extent of shortening was markedly reduced (*p* < 0.001 vs. CP), and the colons appeared less contracted and edematous. These findings indicate that HMP mitigated the structural damage and inflammatory edema typically induced by cisplatin.

### 3.5 HMP improved immune organ indices impaired by cisplatin

The spleen and thymus indices were examined to assess systemic immune function (Figures 2J,K). In healthy mice, both the control and HMP-alone groups showed comparable organ weights, indicating that HMP administration by itself did not interfere with normal immune development. Following cisplatin exposure, a pronounced decline was observed in both spleen and thymus indices compared with the control group (*p* < 0.001 and *p* < 0.0001, respectively), reflecting the strong immunosuppressive effect of chemotherapy. However, when HMP was given together with cisplatin, the values of both indices increased significantly (*p* < 0.001 vs. CP), approaching those seen in the control group. Although not fully normalized, the recovery was substantial compared with the untreated model. These findings suggest that HMP effectively mitigates cisplatin-induced atrophy of immune organs and helps preserve systemic immune competence during chemotherapy.

### 3.6 HMP ameliorated cisplatin-induced histopathological damage in the intestine

**Figure 3:**
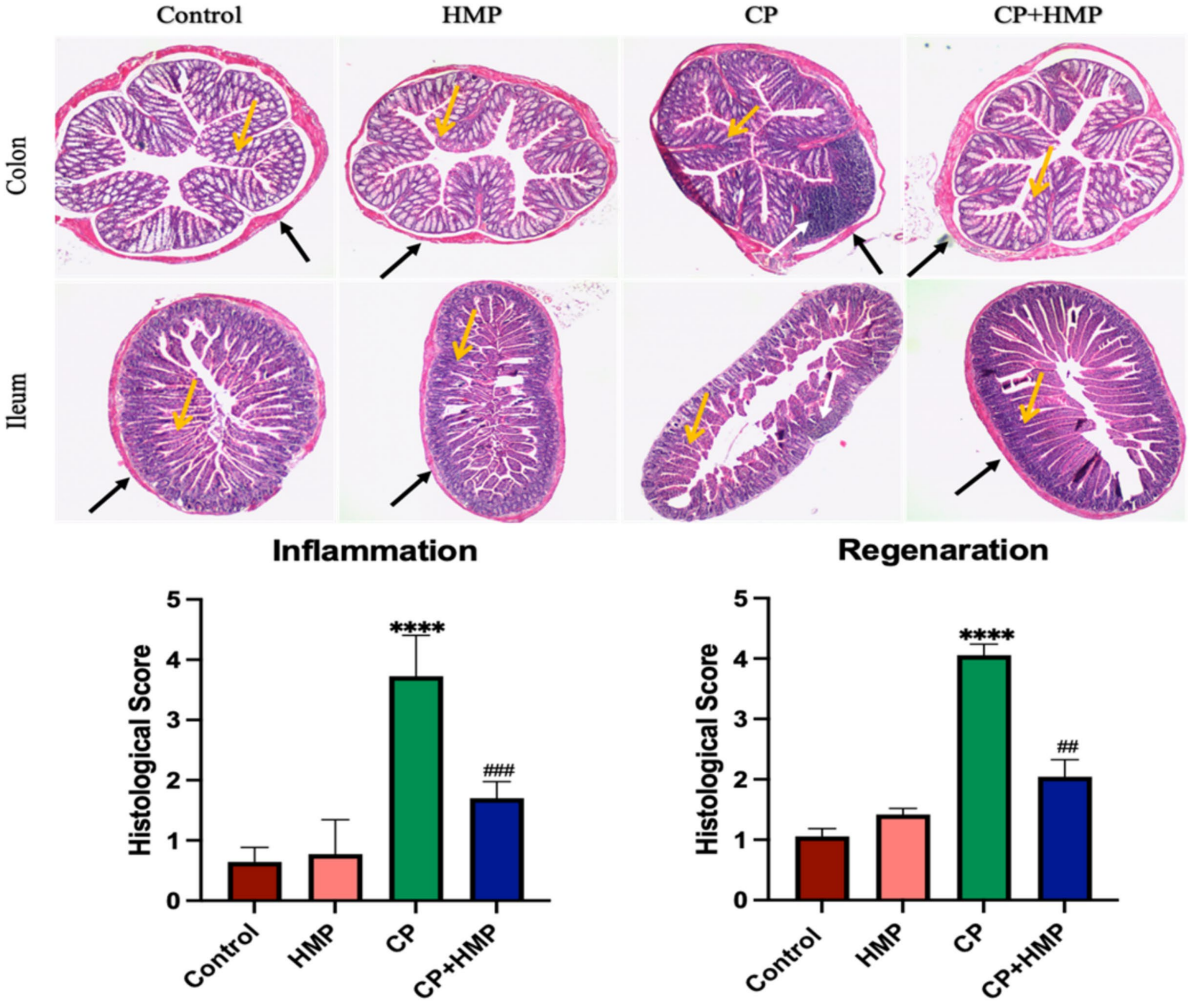
HMP attenuates cisplatin-induced intestinal injury and promotes mucosal repair. Representative H&E images of colon and ileum. CP caused epithelial erosion/ulceration, crypt loss, leukocyte infiltration (black arrows), and crypt distortion (yellow arrows). HMP co-treatment preserved epithelial architecture and reduced inflammation. Bottom panels show semiquantitative histological scoring of inflammation and regeneration. CP increased inflammation and impaired regeneration; HMP significantly reduced inflammation and improved regeneration. Data are mean ± SD; statistical significance vs. control and CP is indicated.

Histopathological evaluation of colon and ileum sections was performed using H&E staining to assess the extent of mucosal injury and the protective effect of HMP (Figure 3). In the control group, both the colon and ileum exhibited intact epithelial architecture with well-organized crypts, abundant goblet cells, and no evidence of inflammatory infiltration. Similarly, mice receiving HMP alone displayed normal tissue morphology, confirming that the polysaccharide had no adverse impact on intestinal structure. In contrast, cisplatin (CP) administration caused pronounced mucosal damage characterized by epithelial disruption, villus blunting, crypt atrophy, goblet cell depletion, and dense inflammatory cell infiltration in the lamina propria. These pathological features are consistent with acute mucositis and barrier breakdown. Importantly, co-administration of HMP with cisplatin markedly mitigated these histological abnormalities. The CP + HMP group showed more continuous epithelial lining, partially restored crypt organization, and a visible reduction in inflammatory cell accumulation compared with the CP group. Quantitative scoring of inflammation and regeneration further supported these findings. CP-treated mice exhibited significantly elevated inflammation and regeneration scores relative to the control group (*p* < 0.001), reflecting severe mucosal injury and impaired repair processes. In contrast, HMP supplementation significantly reduced both scores (*p* < 0.001 vs. CP), indicating substantial recovery of tissue structure and function. Overall, these observations confirm that HMP effectively attenuated cisplatin-induced intestinal mucositis by preserving epithelial integrity, supporting tissue regeneration, and suppressing local inflammation.

### 3.7 HMP preserves intestinal barrier integrity and mucin secretion in cisplatin-induced mucositis

**Figure 4:**
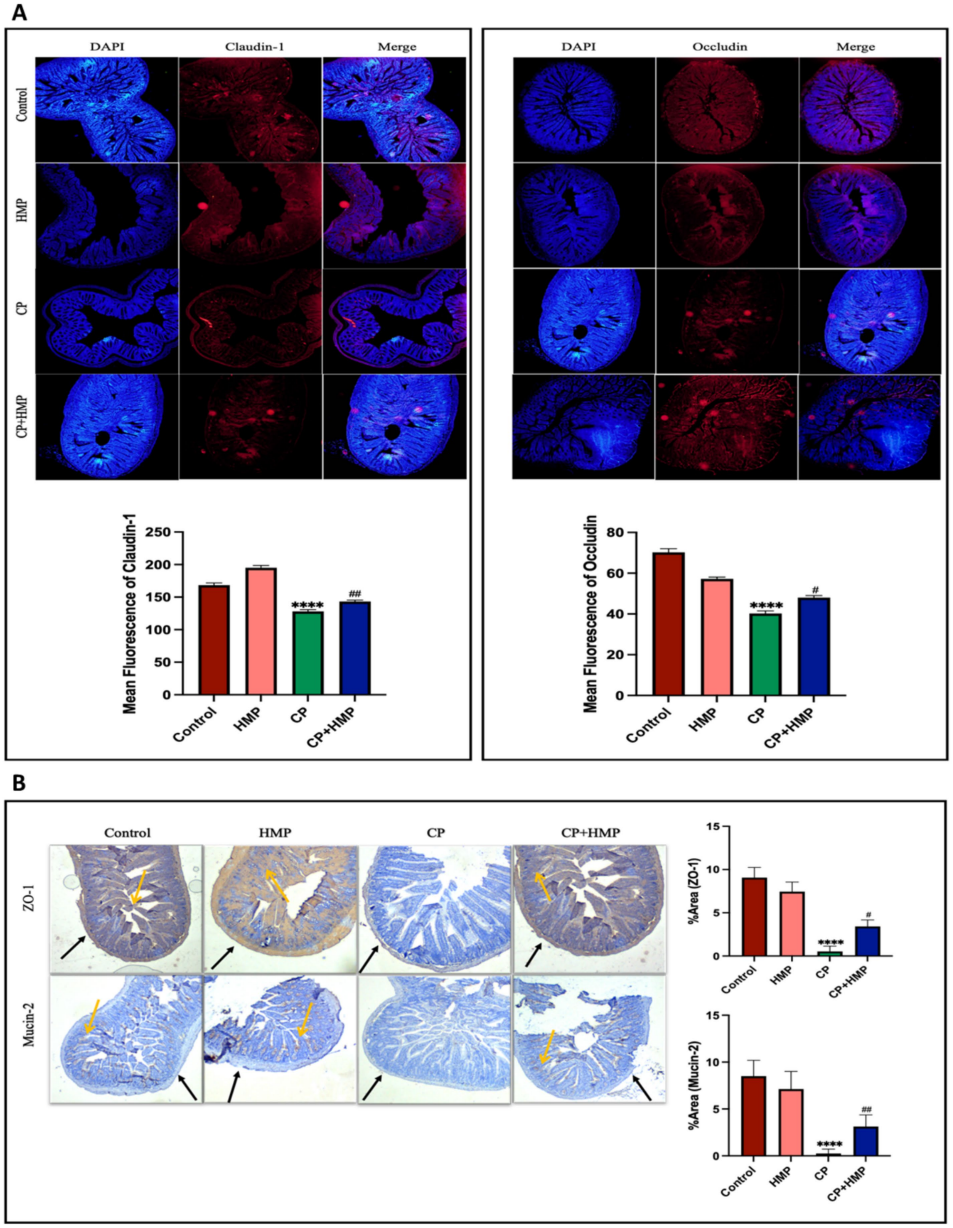
HMP preserves ileal epithelial barrier proteins depleted by cisplatin. **(A)** Immunofluorescence staining of ileum for Claudin-1 (left) and Occludin (right) with DAPI (red). DAPI was used to stain cell nuclei, and the mean fluorescence intensity of Claudin-1 and Occludin was quantified (bottom graphs). CP reduced junctional staining; HMP partially restored intensity. **(B)** Immunohistochemistry for ZO-1 (top) and Mucin-2 (bottom) with arrows highlighting positive staining across the control, HMP, CP, and CP+HMP groups. CP disrupted ZO-1 localization and depleted goblet-cell mucin; HMP improved ZO-1 continuity and increased Mucin-2-positive area. Quantification of ZO-1 and Mucin-2 area is shown in the bar graph on the right. Data are mean ± SD; statistical significance vs. control and CP is indicated.

**Figure 5:**
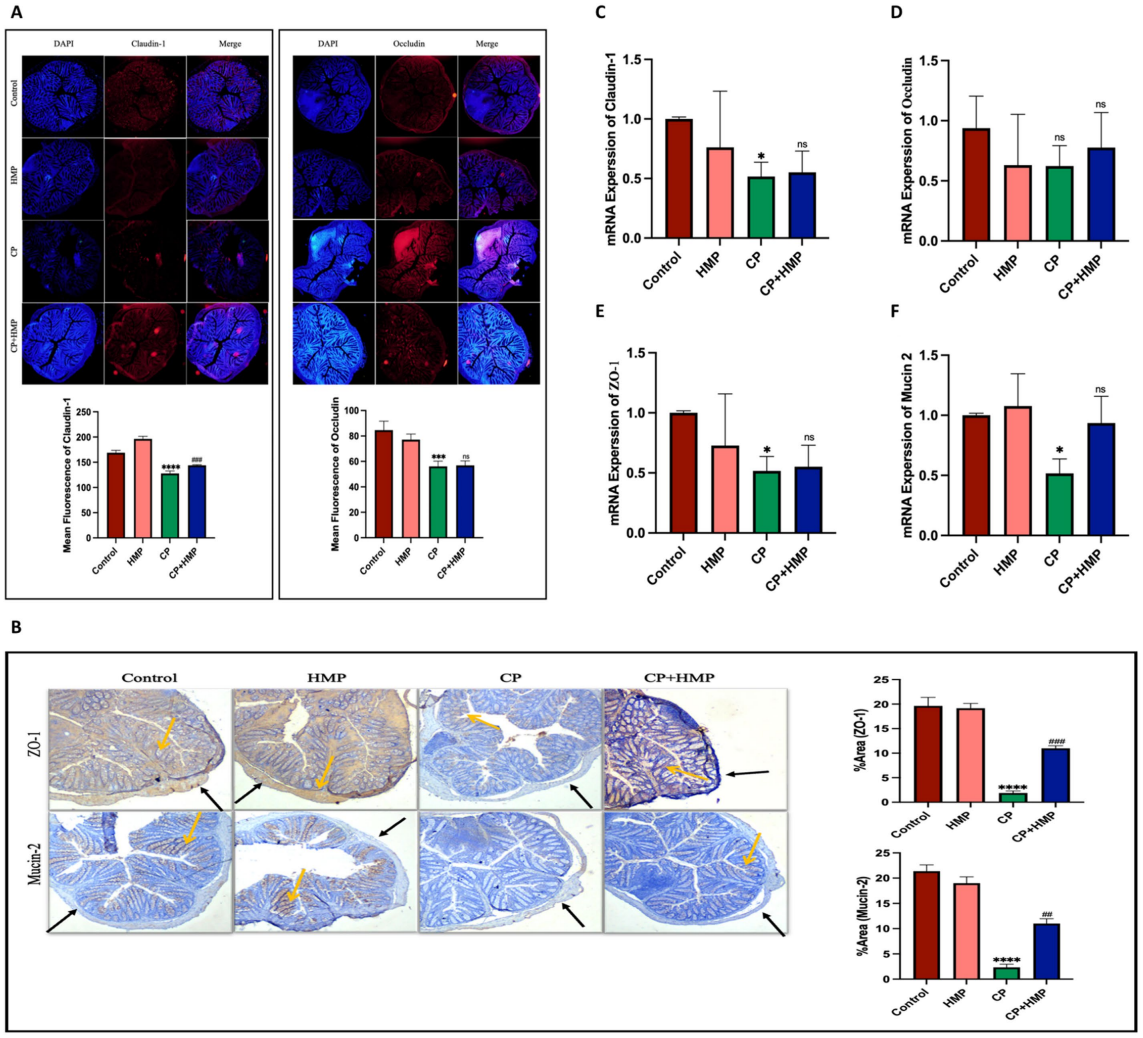
HMP preserves colon epithelial barrier components compromised by cisplatin. **(A)** Immunofluorescence staining of Claudin-1 (left) and Occludin (right) with DAPI (red). DAPI was used to stain cell nuclei. Bar graphs show mean fluorescence intensity of Claudin-1 and Occludin, across Control, HMP, CP, and CP+HMP groups. CP disrupted junctional continuity, while HMP partially restored staining (arrows). **(B)** Immunohistochemical staining of ZO-1 (top) and Mucin-2 (bottom), with arrows indicating positive staining. The percentage of Mucin-2 and ZO-1 positive staining area is represented in the bar graph on the right. HMP improved ZO-1 continuity and increased the Mucin-2-positive area. **(C–F)** qPCR analysis of tight-junction and mucin genes. CP reduced Claudin-1, ZO-1, and Mucin-2 mRNA levels, while Occludin changes were modest. HMP co-treatment partially normalized transcript levels. Data are expressed as mean ± SD, with statistical significance indicated vs. Control and CP groups.

Immunohistochemical (IHC) and immunofluorescence (IF) analyses were performed to assess the impact of cisplatin (CP) and HMP treatment on intestinal barrier integrity by examining the expression of tight junction proteins (Claudin-1, Occludin, ZO-1) and the mucin protein Mucin-2 in both ileum and colon tissues, alongside their respective quantification. In the ileum tissue (Figures 4A,B), tight junction proteins and Mucin-2 exhibited intense, continuous staining in the Control group, indicating intact epithelial architecture. The HMP-only group exhibited similar patterns to the control, suggesting that HMP itself does not alter normal intestinal morphology. In contrast, CP-treated mice displayed disrupted epithelial structure with significantly reduced expression of Claudin-1, Occludin, ZO-1, and Mucin-2 (*p* < 0.0001 vs. Control for all), indicating tight junction breakdown and mucosal damage. However, in the CP+HMP group, the expression of Claudin-1 (*p* < 0.01), Occludin and ZO-1 (*p* < 0.05, respectively) was significantly increased compared to the CP group, with Mucin-2 production also significantly enhanced (*p* < 0.01). These results suggest that CP+HMP treatment improved tight junction protein expression and mucin secretion, indicating a potential restoration of ileal barrier function. In the colon tissue (Figures 5A,B), the control group exhibited strong, continuous staining for Claudin-1, ZO-1, Occludin, and Mucin-2, indicating intact epithelial integrity, which was maintained in the HMP group. In contrast, the CP group showed significant disruption, with reduced expression of Claudin-1, ZO-1, and Mucin-2 (*p* < 0.0001 vs. Control, respectively), and Occludin (*p* < 0.001 vs. Control), reflecting tight junction breakdown. Treatment with CP+HMP restored Claudin-1 and ZO-1 expression to near-Control levels (*p* < 0.001 vs. CP, respectively), and Mucin-2 expression was also significantly restored (*p* < 0.01 vs. CP). However, Occludin expression did not show significant differences compared to the CP group (ns vs. CP). These results suggest that CP+HMP treatment helped restore tight junction protein expression and mucin secretion, indicating potential recovery of intestinal barrier function in both ileum and colon tissues. To further validate these findings, qPCR analysis was performed on colon tissue to measure the relative mRNA expression of *claudin-1*, *occludin*, *zo-1*, and *mucin-2* (Figures 5C–F). In the Control and HMP groups, mRNA levels of all genes remained high and comparable, indicating preserved epithelial homeostasis. In CP-treated mice, *claudin-1* and *zo-1* expression were significantly downregulated (*p* < 0.05 vs. Control), while occludin expression remained unchanged (ns). Mucin-2 expression was also significantly reduced (*p* < 0.05), suggesting compromised mucus barrier integrity. Notably, co-treatment with HMP (CP + HMP) partially restored the mRNA expression of *claudin-1* and *zo-1*, significantly increased *mucin-2* levels (*p* < 0.05), and showed a moderate increase in occludin expression, although the change did not reach statistical significance. These results suggest that HMP preserves intestinal barrier integrity and mucus secretion by enhancing the expression of key barrier-associated genes in colon tissue, promoting epithelial recovery and protecting against cisplatin-induced mucositis.

### 3.8 Anti-inflammatory role of HMP in CP-induced intestinal mucositis

**Figure 6:**
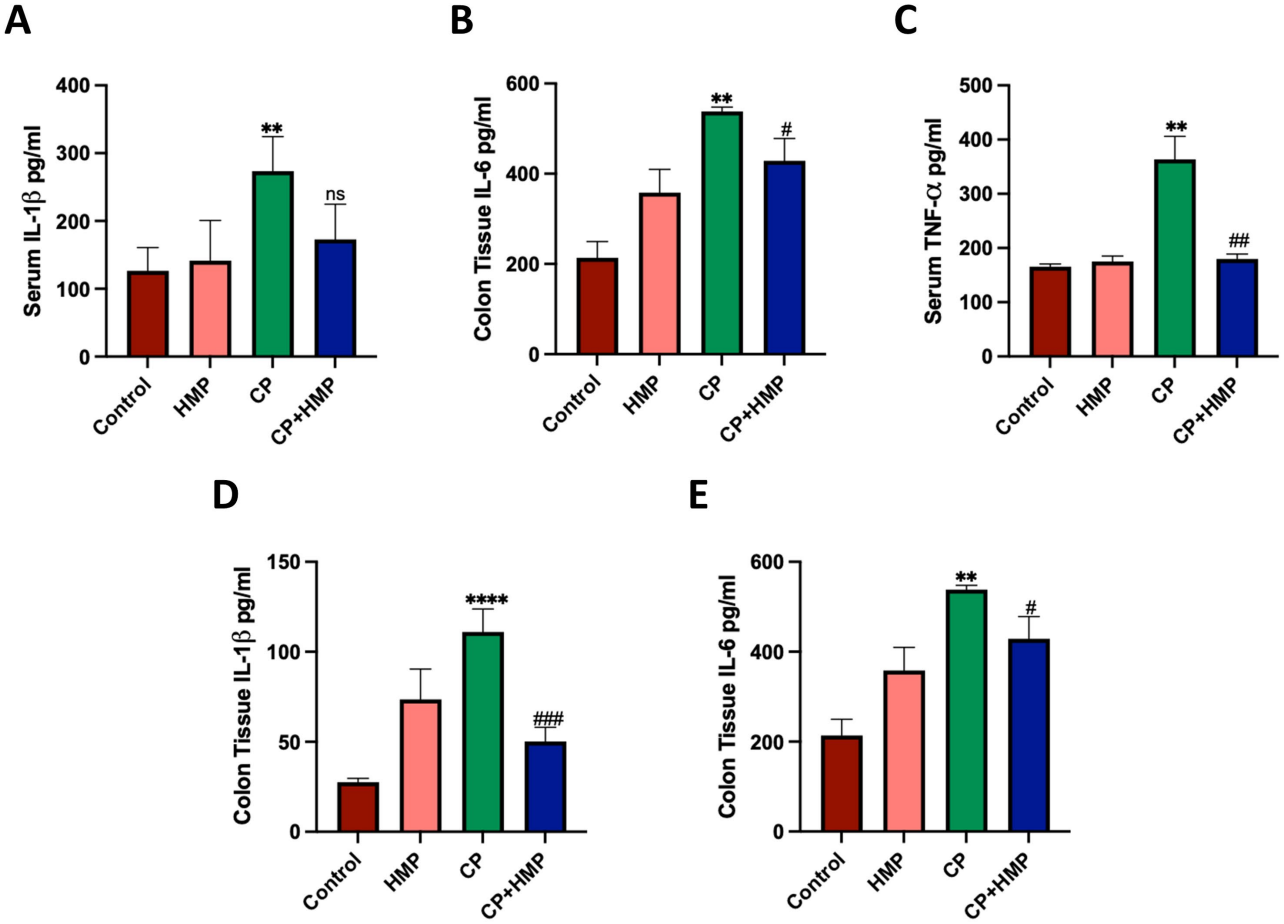
HMP attenuates cisplatin-induced systemic and intestinal inflammation. **(A)** Serum IL-1β levels. **(B)** Serum IL-6 levels. **(C)** Serum TNF-α levels. CP markedly increased all cytokines; HMP co-treatment reduced levels toward baseline. **(D)** Colon IL-1β levels. **(E)** Colon IL-6 levels. CP elevated mucosal cytokines; HMP significantly decreased IL-1β and partially reduced IL-6. Data are mean ± SD; statistical significance vs. control and CP is indicated.

After sacrifice, Serum and tissue cytokine levels were assessed to evaluate the effects of HMP treatment in the context of cisplatin-induced inflammation. Serum concentrations of pro-inflammatory cytokines (IL-1β, IL-6, and TNF-*α*) were quantified (Figures 6A–C). The Control group exhibited normal baseline cytokine levels, while mice treated with HMP alone showed values comparable to controls, indicating that HMP itself did not trigger an inflammatory response. In contrast, CP group markedly elevated serum levels of IL-1β (*p* < 0.01), IL-6 (*p* < 0.05), and TNF-α (*p* < 0.01) compared with the Control group, confirming a strong systemic inflammatory reaction. Co-administration of HMP (CP + HMP) significantly reduced TNF-α levels (*p* < 0.01 vs. CP), while IL-1β and IL-6 showed a downward trend that did not reach statistical significance. In colonic tissue (Figures 6D,E), CP treatment also induced a pronounced increase in IL-1β (*p* < 0.0001) and IL-6 (*p* < 0.01), indicating localized intestinal inflammation. HMP co-treatment markedly suppressed these cytokines (*p* < 0.001 and *p* < 0.01 vs. CP), restoring their levels close to those in the Control and HMP groups. Collectively, these results demonstrate that HMP effectively alleviates both systemic and intestinal inflammation induced by cisplatin, primarily through the downregulation of IL-1β, IL-6, and TNF-α production. This anti-inflammatory property likely contributes to the observed improvements in intestinal morphology and overall recovery in CP-treated mice.

### 3.9 Role of HMP in modulating gut microbiota composition in CP-induced mucositis

**Figure 7:**
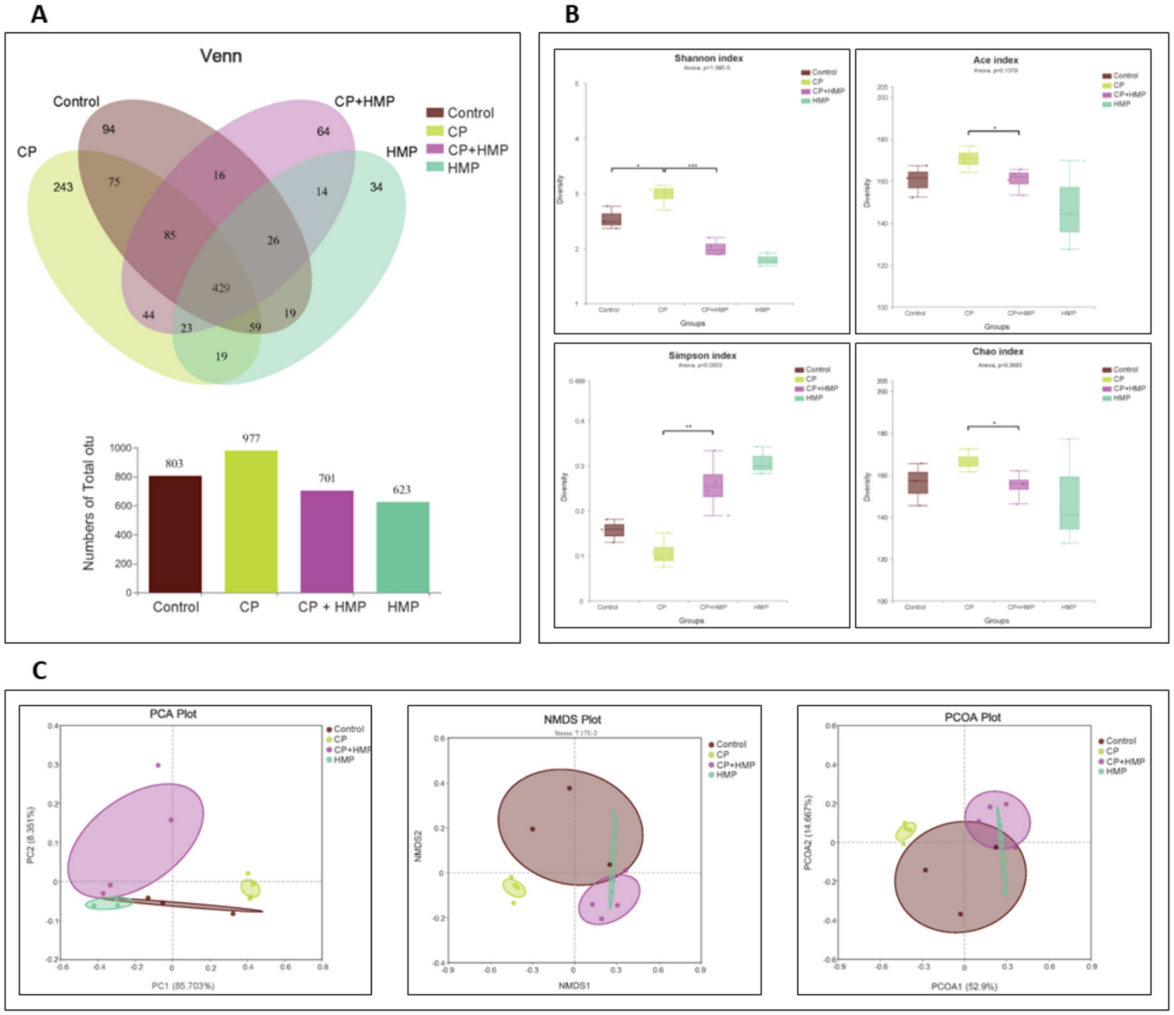
HMP modulates cisplatin-altered gut microbiota diversity and community structure. **(A)** Venn diagram of observed OTUs with total richness bar plot. **(B)** Alpha diversity metrics comparing microbial diversity across groups. CP reduced Shannon index and richness (Ace, Chao) and increased Simpson dominance, indicating loss of evenness. HMP partially restored diversity. **(C)** Beta diversity analysis visualized through principal component analysis (PCA), non-metric multidimensional scaling (NMDS), and principal coordinate analysis (PCoA) plots. Each plot shows the clustering of microbial communities by group, highlighting differences in community structure across the control, CP, CP+HMP, and HMP groups. CP communities clearly separated from control. CP+HMP clustered closer to control, while HMP alone formed a distinct cluster, indicating reshaping of community composition and mitigation of CP-induced dysbiosis.

The Venn diagram in Figure 7A illustrates the overlap of operational taxonomic units (OTUs) among the control, CP, CP+HMP, and HMP groups. A total of 803 OTUs were identified in the control group, 977 in the CP group, 701 in the CP+HMP group, and 623 in the HMP group. Notably, the CP group shared 429 OTUs with the CP+HMP group, suggesting some overlap due to the treatment, while the control group exhibited unique OTUs compared to the treatment groups. The alpha diversity of the gut microbiota was assessed using four different indices: Shannon, Ace, Simpson, and Chao (Figure 7B). The results revealed significant differences in microbial diversity between the groups. The Shannon index showed the control group to have the highest diversity, followed by the CP+HMP group, which was significantly higher than the CP group (*p* < 0.05). The Ace and Chao indices also indicated that the CP group had the lowest richness, whereas the HMP treatment showed a partial recovery in microbial richness compared to CP alone. The Simpson index further confirmed that HMP treatment improved diversity by reducing the dominance of specific taxa. Beta diversity was evaluated using Principal Component Analysis (PCA), Non-metric Multidimensional Scaling (NMDS), and Principal Coordinates Analysis (PCoA) plots (Figure 7C). The PCA plot demonstrated that the control, and treatment groups were well-separated along PC1, indicating distinct microbiota compositions between the groups. Similarly, the NMDS plot showed clear clustering of the CP and HMP groups along NMDS1, reflecting significant differences in microbial community structure. The PCoA plot further supported these findings, with the CP and HMP groups exhibiting different microbial profiles. Overall, the results demonstrate that CP-induced mucositis leads to significant changes in gut microbiota composition, as shown by reduced alpha diversity and distinct beta diversity profiles. HMP treatment partially restores these changes, improving both the diversity and composition of the gut microbiota in CP-treated mice.

### 3.10 LEfSe analysis identifies key taxonomic biomarkers modulated by experimental treatments

**Figure 8:**
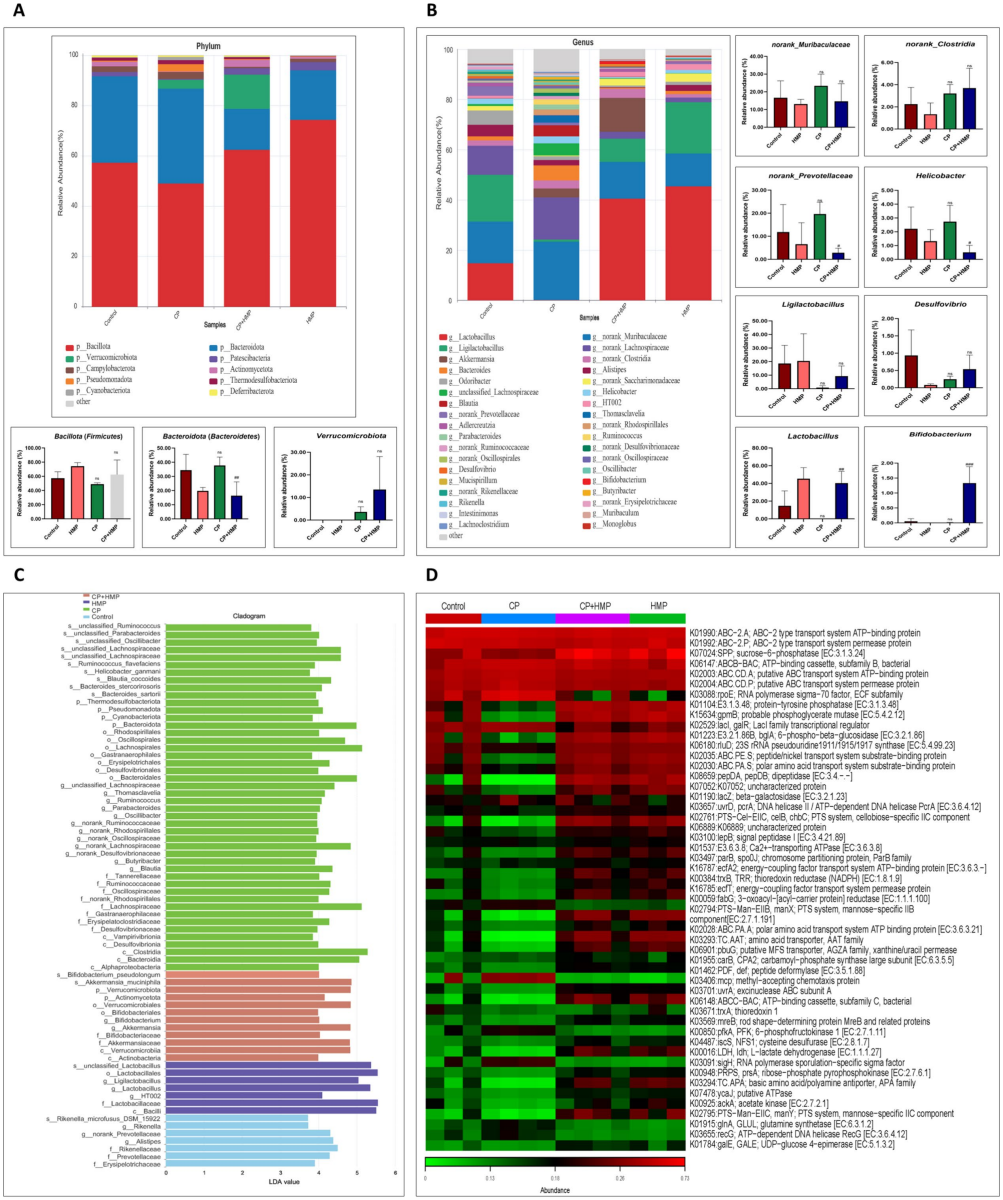
HMP modulates the gut microbiome and functional pathways disrupted by cisplatin. **(A)** Phylum-level bar plots. Inset plots depict the proportions of Bacillota, Bacteroidota, and Verrucomicrobiota across groups (mean ± SD; significance indicated). **(B)** Genus-level microbiome composition. The stacked bars represent the relative abundance of key genera, with individual plots showing the relative abundance of norank_Muribaculaceae, norank_Clostridia, *Helicobacter, Ligilactobacillus, Desulfovibrio, Lactobacillus*, and *Bifidobacterium* in each group. **(C)** Cladogram showing differentially abundant taxa identified through LEfSe analysis. The bars represent the LDA score for each taxon across the groups. **(D)** Heatmap showing the abundance of functional pathways identified by metagenomic analysis, with distinct profiles observed in each treatment group.

LEfSe analysis identifies key taxonomic biomarkers modulated by cisplatin and HMP treatment. Linear discriminant analysis (LDA) coupled with effect size estimation (LEfSe, LDA > 3.0) revealed clear group-specific microbial biomarkers (Figure 8C). The model group exhibited significant enrichment of *Bacteroidota*, *Cyanobacteriota*, and several lineages within *Clostridia*, *Desulfovibrionia*, and *Lachnospiraceae*, including *Oscillibacter*, *Parabacteroides*, *Ruminococcus*, *Helicobacter gammani*, and *Desulfovibrio*. These taxa, with LDA scores exceeding 4.0, are associated with inflammatory and dysbiotic gut states, reflecting cisplatin-induced microbial stress. Conversely, the HMP + cisplatin group showed enrichment of beneficial taxa such as *Akkermansia muciniphila*, *Bifidobacterium pseudolongum*, *Bifidobacteriaceae*, and *Verrucomicrobiota*, all displaying high discriminative power (LDA ≈ 3.5–4.2). These bacteria are known to enhance mucosal integrity and short-chain fatty acid production, suggesting that HMP co-treatment promoted functional recovery of the gut microbiome. The HMP group was characterized by strong enrichment of *Lactobacillus*, *Ligolactobacillus*, *Lactobacillaceae*, and HT002 (LDA ≈ 3.2–3.8), highlighting a probiotic-oriented profile distinct from both Control and Model. In contrast, the Control group primarily featured *Rikenella*, *Alistipes*, *Prevotellaceae*, and *Erysipelotrichaceae* (LDA ≈ 3.0–3.5), Consistent with a balanced microbial community typical of a healthy gut, overall, the LEfSe-based LDA and cladogram analyses confirmed that cisplatin drives a dysbiotic shift enriched in pro-inflammatory and stress-tolerant taxa, whereas HMP administration, particularly in co-treatment, restores beneficial and homeostatic bacterial lineages toward a control-like composition.

### 3.11 HMP attenuates gut microbiota dysbiosis in cisplatin-induced intestinal mucositis

The gut microbial communities were characterized at both the phylum and genus levels across the experimental groups to assess the impact of cisplatin treatment and HMP supplementation on microbial diversity (Figures 8A,B). At the phylum level (Figure 8A; Supplementary Table S3), *Bacillota* (*Firmicutes*) was the most abundant phylum in all groups. The Control group exhibited a relative abundance of 57.42%, and the HMP group, which received only polysaccharide treatment, showed a similar relative abundance of 74.37%. In contrast, the CP group demonstrated a reduction in *Bacillota* (49.08%) compared to both the Control and HMP groups. The CP+HMP group exhibited a marked increase in *Bacillota* (62.61%) compared to the CP group, indicating that HMP supplementation mitigated the decrease in *Bacillota* caused by cisplatin treatment. Additionally, *Bacteroidota* (*Bacteriodetes*) decreased in the CP group (37.38%) compared to the Control group (34.08%), and further declined in the CP+HMP group (16.15%, *p* <0.05) compared to the model group, suggesting that HMP treatment contributed to the reduction in *Bacteroidota* abundance when combined with cisplatin. *Verrucomicrobiota* was notably increased in the CP+HMP group (13.48%) compared to the Control group (0.02%), with no significant difference between the CP+HMP and CP groups. The CP group showed a slight increase in *Verrucomicrobiota* (3.63%) compared to the Control group, but this was substantially lower than in the CP+HMP group, indicating that HMP supplementation may help restore *Verrucomicrobiota* abundance. At the genus level (Figure 8B; Supplementary Table S4), the microbiome in the control group exhibited a stable abundance of beneficial genera, such as norank_Muribaculaceae (16.30%), norank_Lachnospiraceae (11.20%), *Ligilactobacillus* (19.00%), and *Lactobacillus* (15.38%), reflecting a relatively balanced microbial community. *Akkermansia* was present at a low level (0.02%), which is typical for a healthy gut microbiome. Other genera, such as *Bifidobacterium* (0.06%), were also observed, albeit in relatively low proportions. In contrast, the HMP group showed significant shifts, with *Lactobacillus* (45.35%) and *Ligilactobacillus* (20.30%) increasing considerably compared to the control, suggesting that HMP treatment enhanced the abundance of these beneficial genera. Additionally, *Akkermansia* rose to 13.48%, while *Blautia* and *Bifidobacterium* remained low, indicating that HMP treatment may support the growth of beneficial bacteria and contribute to gut health restoration. When comparing the model group to the control group, substantial differences were observed. The abundance of *Lactobacillus* decreased sharply to 0.07% in the model, signaling severe dysbiosis following cisplatin treatment. Similarly, *Ligilactobacillus* dropped to 0.85%, while *Akkermansia* increased to 3.63% in the model group. Additionally, norank_Muribaculaceae increased to 23.23%. These changes highlight the negative impact of cisplatin on the gut microbiome, promoting an environment favoring potentially harmful genera. The increase in *Bacteroides* (5.72%), *Blautia* (4.62%), *Helicobacter* (2.77%), and *Ruminococcus* (2.13%) suggests an imbalance with the overgrowth of specific microbial groups, indicating that cisplatin might alter the microbial landscape by promoting harmful genera. Moreover, *Bifidobacterium* almost vanished, dropping to 0.01%, further indicating the detrimental effects of cisplatin. In comparison, the HMP+CP group showed several beneficial changes relative to the model group. *Lactobacillus* increased to 40.48%, while *Ligilactobacillus* showed a marked recovery, rising to 9.38%. *Akkermansia* rebounded to 13.48%, and Bifidobacterium increased to 1.32% in the treatment group. Additionally, HMP treatment reduced the growth of harmful genera like *Bacteroides* (0.34%), *Blautia* (0.09%), and *Helicobacter* (0.51%). These changes suggest that while cisplatin induces significant dysbiosis and promotes the overgrowth of potentially harmful bacteria, HMP treatment may counterbalance inflammation and support gut health restoration by promoting the growth of beneficial bacteria and suppressing the growth of harmful genera. To further investigate these findings, Linear Discriminant Analysis (LDA) and Effect Size (LEfSe) were performed to evaluate the impact of various treatments on the microbiome composition across the control, model (CP), treatment (CP+HMP) and HMP groups. The analysis identified key microbial biomarkers that were significantly associated with each group (Figure 8C; Supplementary Table S7). The key microbial biomarkers in the control group included *Rikenella* (Family: *Rikenellaceae*, Genus: *Rikenella*, Species: *Rikenella microfusus*) and *Alistipes* (Family: Rikenellaceae, Genus: *Alistipes*, Species: *Alistipes* sp). These taxa, which are known for their positive influence on gut health, were highly abundant, reflecting a balanced and healthy microbiome typical of the unperturbed state. Conversely, the CP model group showed a marked expansion of *Helicobacter* (Family: *Helicobacteraceae*, Genus: *Helicobacter*, Species: *Helicobacter ganmani*) and *Ruminococcus* (Family: Ruminococcaceae, Genus: *Ruminococcus*, Species: *Ruminococcus flavefaciens*), indicative of dysbiosis. This shift toward pro-inflammatory, potentially pathogenic taxa is consistent with the known negative impact of cisplatin on gut health, characterized by inflammation and disruption of the microbiome. The CP+HMP treatment group exhibited significant changes compared to the model group, with an enrichment of *Bifidobacterium pseudolongum* (Class: *Actinobacteria*, Family: *Bifidobacteriaceae*, Genus: *Bifidobacterium*, Species: *Bifidobacterium pseudolongum*) and *Akkermansia muciniphila* (Class: *Verrucomicrobiota*, Family: Akkermansiaceae, Genus: *Akkermansia*, Species: *Akkermansia muciniphila*). Both species are associated with the maintenance of gut integrity and immune modulation, suggesting that HMP treatment restores balance to the microbiome affected by CP. In the HMP group, similar beneficial shifts were observed, with prominent enrichment of *Bifidobacterium pseudolongum* and *Akkermansia muciniphila*, which are known for enhancing gut barrier function and promoting a healthy gut environment. The microbiota composition in this group was largely devoid of the pathogenic taxa observed in the CP group, underscoring the role of HMP in supporting a healthy and stable gut microbiome. The HMP group, similar beneficial shifts were observed, with prominent enrichment of *Lactobacillus* (family: *Lactobacillaceae*) and *Ligilactobacillus* (order: *Lactobacillales*) dominating the microbial composition. However, there was a notable increase in the presence of *Bacilli* (class: *Bacilli*). This shift suggests that HMP alone can effectively modulate the gut microbiome, contributing to a more balanced microbial profile. The treatment likely counteracted some of the dysbiosis induced by the CP model, promoting a healthier gut environment. This finding indicates that HMP treatment has a beneficial impact on the gut microbiome composition, with a potential to alleviate microbial imbalance and restore normal microbiota composition.

### 3.12 Gene expression profiling in response to cisplatin and HMP treatment

Gut microbiota sequencing via KO heatmap analysis was employed to assess alterations in microbial gene expression in response to cisplatin (CP) and HMP treatment (Figure 8D). The analysis revealed reveal significant alterations in gut microbiota protein abundance following cisplatin (CP) and HMP treatments. Cisplatin treatment led to a notable downregulation in several key microbial proteins, including those involved in transport and metabolic processes. Specifically, the abundance of ABC-type transport system proteins, such as K01992: ABC-2. P and K02003: ABC. CD. A, was significantly reduced, suggesting an impairment in microbial transport activities, likely due to the toxic effects of the drug on the microbiota. Similarly, enzymes like K07024: SPP (sucrose-6-phosphatase), involved in carbohydrate metabolism, showed reduced abundance, indicating a decline in microbial energy production. Additionally, while proteins associated with DNA repair, such as K03657:uvrD (DNA helicase), exhibited only minimal changes, their reduced expression suggests that cisplatin-induced DNA damage might hinder microbial DNA repair processes. However, the combined treatment of CP+HMP restored several of these disrupted functions. Notably, K07024: SPP showed a marked increase in abundance, rising from 0.302 (CP) to 0.665 (CP+HMP), suggesting that HMP can counteract the suppression of carbohydrate metabolism induced by cisplatin. Furthermore, K02003: ABC. CD. A (putative ABC transport system ATP-binding protein) and K03657:uvrD (DNA helicase) also exhibited increased abundance in the CP+HMP group, reflecting a restorative effect of HMP on microbial transport and DNA repair systems. These changes suggest that HMP helps restore disrupted microbial functions, likely due to its restorative properties that mitigate the oxidative damage caused by cisplatin, thereby reducing the need for excessive DNA repair and stress responses. In comparison, HMP treatment alone resulted in increased abundance of transport proteins and metabolic enzymes, such as K07024: SPP, which further underscores HMP’s role in enhancing microbial energy production and stability. This increase in protein abundance indicates that HMP may enhance microbial functions related to metabolism, transport, and DNA repair compared to the control group, suggesting its potential to promote overall microbiota health. These findings collectively suggest that HMP exerts a protective and restorative role in the gut microbiota, mitigating cisplatin-induced dysbiosis by restoring essential microbial functions related to transport, metabolism, and DNA repair. Thus, HMP may hold potential as a therapeutic intervention to preserve gut microbiota health during chemotherapy treatment.

## 4 Discussion

Cisplatin (CP), a widely used chemotherapeutic agent, remains effective but is limited by high cytotoxicity and significant side effects, including acute kidney injury, liver dysfunction, bone marrow suppression, gastrointestinal injury, peripheral neuropathy, pulmonary toxicity, reproductive damage, and ototoxicity. These toxicities are primarily mediated by oxidative stress, inflammation, apoptosis, and organ-specific damage (27), particularly in the gastrointestinal system (28).

Intestinal mucositis is a common complication characterized by inflammation of the mucosal lining of the digestive tract, leading to structural, functional, and immunological alterations, (29). Despite increasing understanding of its toxic mechanisms, effective protective strategies remain limited. Recent studies have highlighted mushroom polysaccharides for their immunomodulatory and anti-inflammatory properties and their ability to modulate the gut microbiota (30, 31). These compounds show potential to reduce inflammation, promote tissue repair, and enhance gastrointestinal protection, supporting their use as promising adjuncts to cisplatin therapy.

Structurally, *Hypsizygus marmoreus* polysaccharide extract (HMP) was characterized as a heteropolysaccharide with high molecular weight and a high glucose content, predominantly composed of glucose, galactose, mannose, and other monosaccharides. Similarly, exopolysaccharides (EPS) isolated from *C. sinensis* Cs-HK1, a mycelial fermentation product, are composed of glucose, mannose, and galactose, and with a high molecular weight, showed beneficial activity in modulating the intestinal microbiota (32). Additionally, glucose enhances the growth of beneficial bacteria and short-chain fatty acid production, xylose supports probiotic activity, and galactose inhibits harmful bacteria, collectively improving gut health (31). The ABTS^+^ radical-scavenging assay demonstrated that HMP possesses significant, concentration-dependent antioxidant activity, with higher concentrations approaching complete radical inhibition. These results are in line with previous reports on mushroom polysaccharides, wherein ABTS^+^ scavenging increased with concentration, indicating strong *in vitro* antioxidant potential (33). The antioxidant activity of polysaccharides is influenced not only by their total content but also by structural features such as monosaccharide composition, glycosidic linkages, and functional groups, with different molecular weights and compositions resulting in distinct radical-scavenging abilities (34). Together, these structural and functional properties of HMP support its multifunctional role, including antioxidant, anti-inflammatory, and gut-modulatory effects, which likely contribute to protection against cisplatin-induced intestinal toxicity by mitigating oxidative stress, preserving epithelial integrity, and promoting a healthy gut microbiota (35).

In a previous study, chemotherapy-induced gastrointestinal injury was observed at a 10 mg/kg dose of cisplatin. However, a higher dose resulted in significant animal mortality. These findings align with our approach of using the 6 mg/kg dose to induce mucositis and evaluate the effect of HMP on its symptoms (36).

In the model group the results are consistent with the well-documented systemic toxicity of cisplatin. CP targets rapidly proliferating cells, making the gastrointestinal epithelium highly vulnerable to its toxic effects. This leads to gastrointestinal issues, including nausea, vomiting, diarrhea, and impaired intestinal barrier function (37). Cisplatin-induced damage further impairs water absorption and disrupts enzyme function, contributing to diarrhea (38). In this study, HMP administration alongside cisplatin mitigated these effects, preserving weight, food intake, reducing diarrhea, and colon shortening. Similarly, BO treatment has been shown to mitigate the effects of 5-FU-induced intestinal mucositis, preserving weight, food intake, reducing diarrhea, and colon shortening (39). Additionally, HMP administration preserved immune function, as demonstrated by the maintenance of thymus and spleen indices, suggesting its potential to counteract cisplatin-induced immunosuppression. These results are consistent with prior research, which observed that *Lactobacillus rhamnosus* (LBS) treatment also preserved thymus and spleen indices in control and treatment groups, indicating its protective role against cisplatin-induced immunosuppression (24).

Inflammation plays a critical role in defending the body against pathogens and facilitating tissue repair. However, excessive or prolonged inflammation can cause harm, contributing to conditions such as autoimmune disorders and tissue damage. In the context of cisplatin-induced intestinal mucositis, inflammation exacerbates tissue injury (29). Toll-like receptors (TLRs) are crucial in regulating intestinal innate immunity, with TLR4 specifically mediating immune and inflammatory responses to microbial infection. Activation of TLR4 recruits MyD88 during the disruption of intestinal homeostasis, initiating the NF-κB signaling cascade, increasing pro-inflammatory cytokines, and worsening the inflammatory response, further damaging the mucosa (40). Cytokines, including interleukins and chemokines released by immune and damaged epithelial cells, drive the inflammatory response, promoting local and systemic inflammation, and potentially offering protective effects (41). Pro-inflammatory cytokines such as IL-1β, IL-6, TNF-*α*, and NF-κB are elevated after cisplatin treatment, worsening mucositis (42). HMP treatment reduced these cytokine levels, suggesting its potential to counteract inflammation and protect the intestinal barrier (43, 44). Additionally, inflammasome activation and oxidative stress play a significant role in chemotherapy-induced mucositis, further contributing to cytokine release (41). HMP’s antioxidant properties also mitigate oxidative stress, supporting its anti-inflammatory effects. Similarly, Sonis (7) outlined a five-step model for chemotherapy-induced mucositis (IM), with key events involving reactive oxygen species, inflammation, and apoptosis. NF-κB activation upregulates TNF-α and COX-2, contributing to mucosal toxicity. The study showed that HPW could alleviate 5-FU-induced downregulation of these molecules, reduce IL-1β levels, and increase IL-10 expression, suggesting HPW’s potential in modulating inflammation in IM (7).

Furthermore, intestinal barrier integrity is essential for gastrointestinal function, and chemotherapy, including cisplatin, impairs intestinal permeability, contributing to inflammation (4). Histological analysis of the colon and ileum showed significant damage after cisplatin treatment, including epithelial disruption and goblet cell depletion. HMP treatment restored intestinal architecture, reduced tissue edema, and alleviated colonic shortening, supporting its role in maintaining intestinal barrier integrity. Similar protective effects on histological damage have been observed in *5-Fu*-induced intestinal mucositis, where polysaccharides from *Aconitum carmichaelii* Debx reduce mucosal injury through anti-inflammatory activity and regulation of intestinal microbiota metabolism in mice (45).

Tight junction (TJ) proteins, such as Claudin-1, ZO-1, and Occludin, form a near leak-proof intercellular seal that preserves intestinal epithelial integrity by connecting adjacent cells and maintaining the actin cytoskeleton (46, 47). Disruption of these proteins leads to increased permeability and is a common consequence of chemotherapy-induced mucositis (48). TJs also act as a physical barrier, preventing the passage of harmful substances such as bacteria, toxins, and digestive enzymes, and their disruption promotes intestinal inflammation (49). In this context, HMP treatment has been shown to preserve tight junction proteins, reduce permeability, and mitigate inflammation (47, 50). Additionally, mucosal damage plays a key role in the acute symptoms associated with anticancer treatment, and ongoing gastrointestinal symptoms after treatment suggest potential long-term damage to gastrointestinal innervation (51). However, HMP treatment has been shown to upregulate Mucin-2, a critical component of the intestinal barrier involved in mucosal defense and gut homeostasis (52). This suggests that HMP helps restore intestinal barrier integrity and mitigates cisplatin-induced damage. Similar protective effects have been observed with other natural compounds that preserve tight junction proteins and Mucin-2 expression in chemotherapy-induced mucositis. Comparable results have been reported for *Poria cocos*, which alleviates cisplatin-induced gastrointestinal damage (53). These findings underscore the potential of HMP to mitigate chemotherapy-induced gastrointestinal injury and protect the intestinal barrier.

Disruption of the balance between the gut microbiota and the host has been implicated in various diseases associated with gut barrier dysfunction (54). Maintaining a stable gut microbiota is, therefore, a potential strategy to prevent chemotherapy-induced mucositis. Among the approximately 52 known bacterial phyla, 5 to 7 typically inhabit the mammalian gut, with *Bacteroidetes* and *Firmicutes* dominating, while *Proteobacteria*, *Actinobacteria*, and *Verrucomicrobia* are less prevalent (55). In this study, the microbiota analysis revealed a significant decrease in *Firmicutes* in the model group, consistent with the gut microbial profile observed in cancer patients who develop gastrointestinal mucositis following chemotherapy (56). In the control group, *Rikenella*, *Alistipes*, and *Prevotellaceae* were present, reflecting a healthy gut microbiota. *Rikenella*, a beneficial probiotic, has been shown to reduce intestinal inflammation, support tight junction formation, and strengthen the intestinal barrier. Its metabolites, including succinic, propionic, and acetic acids, promote a healthier gut environment and increase microbial diversity, with *Rikenella* abundance negatively correlated with diarrhea severity (57).

Following cisplatin treatment, the microbiota analysis revealed significant dysbiosis, including a reduction in beneficial *Lactobacillus* and an increase in harmful taxa like *norank_Muribaculaceae* and *norank_Lachnospiraceae*, consistent with known chemotherapy-induced disturbances (58). Additionally, previous studies have highlighted the role of *Blautia* in chemotherapy-induced dysbiosis (59), and our results confirmed this with *Ruminococcaceae* and *Ruminococcus* being positively correlated with 5-HT levels, emphasizing the role of gut microbiota in chemotherapy-related side effects (58).

Specifically, HMP co-treatment with cisplatin enriched beneficial taxa, including *Akkermansia muciniphila* and *Bifidobacterium pseudolongum*. These bacteria are known to enhance mucosal integrity and short-chain fatty acid production, suggesting that HMP co-treatment promoted functional recovery of the gut microbiome. While reducing the abundance of pro-inflammatory taxa such as *Helicobacter* and *Ruminococcus*. This microbial shift indicates that HMP not only mitigates cisplatin-induced dysbiosis but also promotes a more balanced and health-supporting microbiome. Moreover, Linear Discriminant Analysis (LDA) and Effect Size (LEfSe) analysis further supported these findings, identifying key taxonomic biomarkers modulated by HMP treatment. This aligns with findings that *Poria cocos* and its components can reduce intestinal injury and modulate gut microbiota, offering a protective effect against dysbiosis and inflammation (53). Notably, HMP supplementation did not induce toxicity or harmful changes in the microbiota when administered alone, confirming its safety.

Polysaccharides from *H. marmoreus* act as non-digestible prebiotics that protect the intestine through antioxidant, anti-inflammatory, mucosal-protective, and microbiota-modulating activities (60). Chemotherapy-induced intestinal injury is driven by oxidative stress and inflammation, resulting in tight junction disruption, mucin depletion, cytokine overproduction, and microbial dysbiosis (29). Consistent with this, cisplatin caused loss of barrier proteins (Claudin-1, ZO-1, Occludin), reduced Mucin-2, elevated IL-1β, IL-6, and TNF-*α*, and altered microbiota composition, including depletion of *Lactobacillus* and *Bifidobacterium* and expansion of *Helicobacter* and *Ruminococcus*. HMP co-treatment mitigated these effects by restoring microbial diversity and beneficial taxa (*Akkermansia muciniphila and Bifidobacterium pseudolongum*), recovering microbial metabolic functions, suppressing inflammatory cytokines, and improving barrier protein and mucin expression. These findings suggest a comprehensive mechanism by which HMP protects against cisplatin-induced mucositis. HMP likely attenuates oxidative stress-driven inflammation and preserves epithelial and mucosal integrity. Additionally, HMP may influence inflammation-related signalling pathways, including ROS-TLR4/NF-κB, although this requires further experimental validation.

In conclusion, this study highlights the protective effects of HMP against cisplatin-induced toxicity, including alleviating systemic symptoms, preserving immune function, maintaining intestinal barrier integrity, reducing inflammation, and modulating gut microbiota composition. These effects are likely mediated by HMP’s ability to enhance epithelial regeneration, suppress oxidative stress, and modulate inflammatory pathways. The findings suggest that HMP may serve as a valuable complementary therapy to improve chemotherapy tolerance and promote gut health in patients with cancer. However, further research is necessary to clarify the molecular mechanisms behind these protective effects and to fully understand HMP’s therapeutic potential. Identifying the specific signaling pathways involved will be crucial to realizing the full scope of HMP’s benefits.

## 5 Conclusion

This study demonstrated that *Hypsizygus marmoreus* polysaccharides (HMP), with a high molecular weight, rich in glucose and galactose, and exhibiting strong *in vitro* antioxidant activity, effectively mitigates cisplatin-induced intestinal mucositis by preserving epithelial barrier integrity, reducing inflammation, and restoring gut microbiota balance. HMP treatment alleviated body weight loss, diarrhea, and immune organ atrophy, while enhancing tight junction protein expression, mucin secretion, and microbial diversity.

These protective effects were associated with decreased oxidative stress, suppression of pro-inflammatory cytokines, and modulation of cisplatin-induced dysbiosis, highlighting the multifaceted mechanisms of HMP.

Overall, HMP represents a promising adjuvant strategy for reducing chemotherapy-induced gastrointestinal toxicity and improving treatment tolerance. Further preclinical and clinical studies are warranted to validate its therapeutic potential and elucidate the underlying molecular pathways.

## Data Availability

The raw data supporting the conclusions of this article will be made available by the authors, without undue reservation.
