# Correction: *Hypsizygus marmoreus* polysaccharides protect against cisplatin-induced intestinal mucositis via modulation of gut microbiota, inflammation, and intestinal barrier function

**DOI:** 10.3389/fnut.2026.1903275

**Published:** 2026-06-23

**Authors:** Frontiers Production Office

**Affiliations:** Frontiers Media SA, Lausanne, Switzerland

**Keywords:** 16S rRNA sequencing, cisplatin, gut microbiota dysbiosis, *Hypsizygus marmoreus*, intestinal mucositis, polysaccharides, systemic inflammation

In the published article, corresponding author Liang Wang was assigned the wrong email address. The Correspondence section has been updated with Liang Wang’s correct email address: wangliang@dmu.edu.cn

Liang Wang's affiliation “Stem Cell Clinical Research Center, National Joint Engineering Laboratory, Regenerative Medicine Center, The First Affiliated Hospital of Dalian Medical University, Dalian, Liaoning Province, China” was omitted in the published article. This affiliation has been added as affiliation seven. Liang Wang's assigned affiliation has been updated from six to seven.

The copyright statement did not reflect the author list in the published article. The author order in the copyright statement has been updated to match the author list:

“© 2026 Abusidu, Alioui, Al-tibi, Alwayli, Ali, Rahman, Farooqui, Atta, Feng, Lu and Wang. This is an open-access article distributed under the terms of the Creative Commons Attribution License (CC BY). The use, distribution or reproduction in other forums is permitted, provided the original author(s) and the copyright owner(s) are credited and that the original publication in this journal is cited, in accordance with accepted academic practice. No use, distribution or reproduction is permitted which does not comply with these terms.”

The article citation did not reflect the author list in the published article. The author order in the article citation has been updated to match the author list:

“Abusidu M, Alioui Y, Al-tibi L, Alwayli D, Ali S, Rahman MU, Farooqui NA, Atta A, Feng B, Lu S, Wang L. *Hypsizygus marmoreus* polysaccharides protect against cisplatin-induced intestinal mucositis via modulation of gut microbiota, inflammation, and intestinal barrier function. *Front. Nutr*. (2026) 13:1774118. doi: 10.3389/fnut.2026.1774118”

The original version of this article has been updated.

