# Supplementary material for: Hypsizygus marmoreus polysaccharides protect against cisplatin-induced intestinal mucositis via modulation of gut microbiota, inflammation, and intestinal barrier function
Source: Front Nutr. 2026 Apr 10;13:1774118. doi: 10.3389/fnut.2026.1774118 (PMC13108610; doi:10.3389/fnut.2026.1774118)
Supplement: Supplementary file 1 [file Datasheet_1.docx]

***Hypsizygus marmoreus* Polysaccharides Protect Against Cisplatin-Induced Intestinal Mucositis via Modulation of Gut Microbiota, Inflammation, and Intestinal Barrier Function**

**Mohammed Abusidu ^1,^ †, Yamina Alioui ^1,2,^ †, Shuming Lu ^3,^ †, Lara Al-tibi ^4^, Dhafer Alwayli ^5^, Sharafat Ali ^6^, Mujeeb Ur Rahman ^1^, Nabeel Ahmed Farooqui ^1^, Aamna Atta ^1^, Bin Feng ^1^ and Liang Wang ^3^***

^1^ Department of Biotechnology, College of Basic Medical Science, Dalian Medical University, Dalian, Liaoning Province, 116011, P.R. China.

^2^The Marine Biomedical Research Institute of Guangdong Zhanjiang, School of Ocean and Tropical Medicine, Guangdong Medical University, Zhanjiang, Guangdong, 524023, China.

^3^ Department of Gastroenterology, First Affiliated Hospital of Dalian Medical University, Dalian, Liaoning Province, 116011, P.R. China.

^4^ Department of Laboratory Medicine Sciences, College of Medicine Faculty of Medical Sciences, Al-Aqsa University, Gaza 4051, Palestine.

^5^ Department of Pathogen Biology and Microecology, College of Basic Medical Sciences, Dalian Medical University, Dalian, Liaoning Province, 116011, P.R. China.

^6^ Department of Biochemistry and Molecular Biology, College of Basic Medical Science, Dalian Medical University, Dalian, Liaoning Province, 116011, P.R. China.

†These authors contributed equally to this work.

***Correspondence authors**: Liang Wang.

**Supplementary Tables:**

**Table S1.** Antibodies used for immunofluorescence (IF) and immunohistochemistry (IHC).

| **Antibody target** | **Antibody type** | **Antibody dilution** | **Catalog Number** | **Company** |
| --- | --- | --- | --- | --- |
| Mucin-2 | Polyclonal | 1:1000 | 27675-1-AP | Proteintech |
| ZO-1 | Polyclonal | 1:1000 | 21773-1-AP | Proteintech |
| Claudin-1 | Polyclonal | 1:1000 | 13050-1-AP | Proteintech |
| Occludin | Polyclonal | 1:400 | 27260-1-AP | Proteintech |

**Table S2.** List of primers used to measure mRNA expression levels.

| **Gene** | **Forward Primer 5ʹ to 3ʹ** | **Reverse Primer 5ʹ to 3ʹ** |
| --- | --- | --- |
| β-actin | ATCGCTGCGCTGGTCG | GTCCTTCTGACCCATTCCCA |
| Mucin-2 | CCGGATCTATGCCGTTGCTA | TCCAGGTGGGTATCGAGTGT |
| Claudin-1 | GCTGGGTTTCATCCTGGCTTCTC | CCTGAGCGGTCACGATGTTGTC |
| Occludin | TGGCAAGCGATCATACCCAGAG | CTGCCTGAAGTCATCCACACTC |
| ZO-1 | GTTGGTACGGTGCCCTGAAAGA | GCTGACAGGTAGGACAGACGAT |

**Table S3.** Percentage Composition of Bacterial Phylum Across the Different Experimental Groups.

| **Phylum** | **Control (%)** | | **HMP (%)** | **Model (%)** | **CP+HMP (%)** |
| --- | --- | --- | --- | --- | --- |
| *Bacillota* (*Firmicutes*) | 57.41854499 | 74.37206391 | | 49.07781149 | 62.60972706 |
| *Bacteroidota* | 34.07917908 | 19.76760439 | | 37.38044436 | 16.14668528 |
| *Verrucomicrobiota* | 0.01645248 | 0.009723666 | | 3.633160419 | 13.4785513 |
| *Patescibacteria* | 1.86339567 | 3.218533307 | | 0.331212685 | 2.612467564 |

**Table S4.** Percentage Composition of Bacterial Genus Across the Different Experimental Groups.

| **Genus** | **Control (%)** | **HMP (%)** | **Model (%)** | **CP+HMP (%)** |
| --- | --- | --- | --- | --- |
| *Lactobacillus* | 15.38063 | 45.34996 | 0.071645 | 40.48361 |
| *norank_Muribaculaceae* | 16.29831 | 13.1464 | 23.22834 | 14.41375 |
| *Ligilactobacillus* | 19.00322 | 20.29876 | 0.846824 | 9.383644 |
| *norank_Lachnospiraceae* | 11.20231 | 2.032854 | 19.72321 | 2.834705 |
| *Akkermansia* | 0.016452 | 0.009724 | 3.63316 | 13.47855 |
| *norank_Clostridia* | 2.227178 | 1.327888 | 3.184105 | 3.718945 |
| *Odoribacter* | 5.768727 | 1.325457 | 1.580503 | 0.699256 |
| *Alistipes* | 4.623147 | 2.371359 | 2.145444 | 0.205966 |
| *Bacteroides* | 1.556283 | 1.281093 | 5.724577 | 0.342991 |
| *Candidatus_Saccharimonas* | 1.860349 | 3.207594 | 0.328472 | 2.603904 |
| *Helicobacter* | 2.137604 | 1.302971 | 2.779681 | 0.512131 |
| *norank_Prevotellaceae* | 3.63417 | 0.614414 | 1.015954 | 0.106623 |
| *Limosilactobacillus* | 1.031631 | 2.255283 | 0.027405 | 1.929484 |
| *Blautia* | 0.198648 | 0.039502 | 4.621317 | 0.095489 |
| *Adlercreutzia* | 1.430147 | 0.554249 | 0.111187 | 1.055093 |
| *Thomasclavelia* | 0.01889 | 0.004254 | 2.98248 | 0.001285 |
| *norank_Oscillospiraceae* | 0.77022 | 0.190827 | 1.622394 | 0.167427 |
| *norank_Rhodospirillales* | 0.024983 | 0.000608 | 2.432025 | 0.013703 |
| *Parabacteroides* | 0.322347 | 0.129446 | 1.899188 | 0.101912 |
| *Ruminococcus* | 0.073122 | 0.0079 | 2.129784 | 0.023123 |
| *norank_Ruminococcaceae* | 0.399734 | 0.127623 | 1.0461 | 0.419211 |
| *norank_Desulfovibrionaceae* | 0.248006 | 0.006685 | 1.342468 | 0.17385 |
| *norank_Oscillospirales* | 0.036561 | 0.017016 | 1.593814 | 0.022695 |
| *norank_Erysipelotrichaceae* | 1.03285 | 0.159225 | 0.189097 | 0.12161 |
| *Bifidobacterium* | 0.057888 | 0.004254 | 0.011354 | 1.316297 |
| *Butyribacter* | 0.137713 | 0.001215 | 1.163159 | 0.001285 |
| *Oscillibacter* | 0.163306 | 0.0079 | 1.096995 | 0.023551 |
| *unclassified_Lachnospiraceae* | 0.519776 | 0.052872 | 1.873348 | 0.112189 |
| Other | 9.825787 | 4.172668 | 11.59597 | 5.637723 |

**Supplementary Figures:**


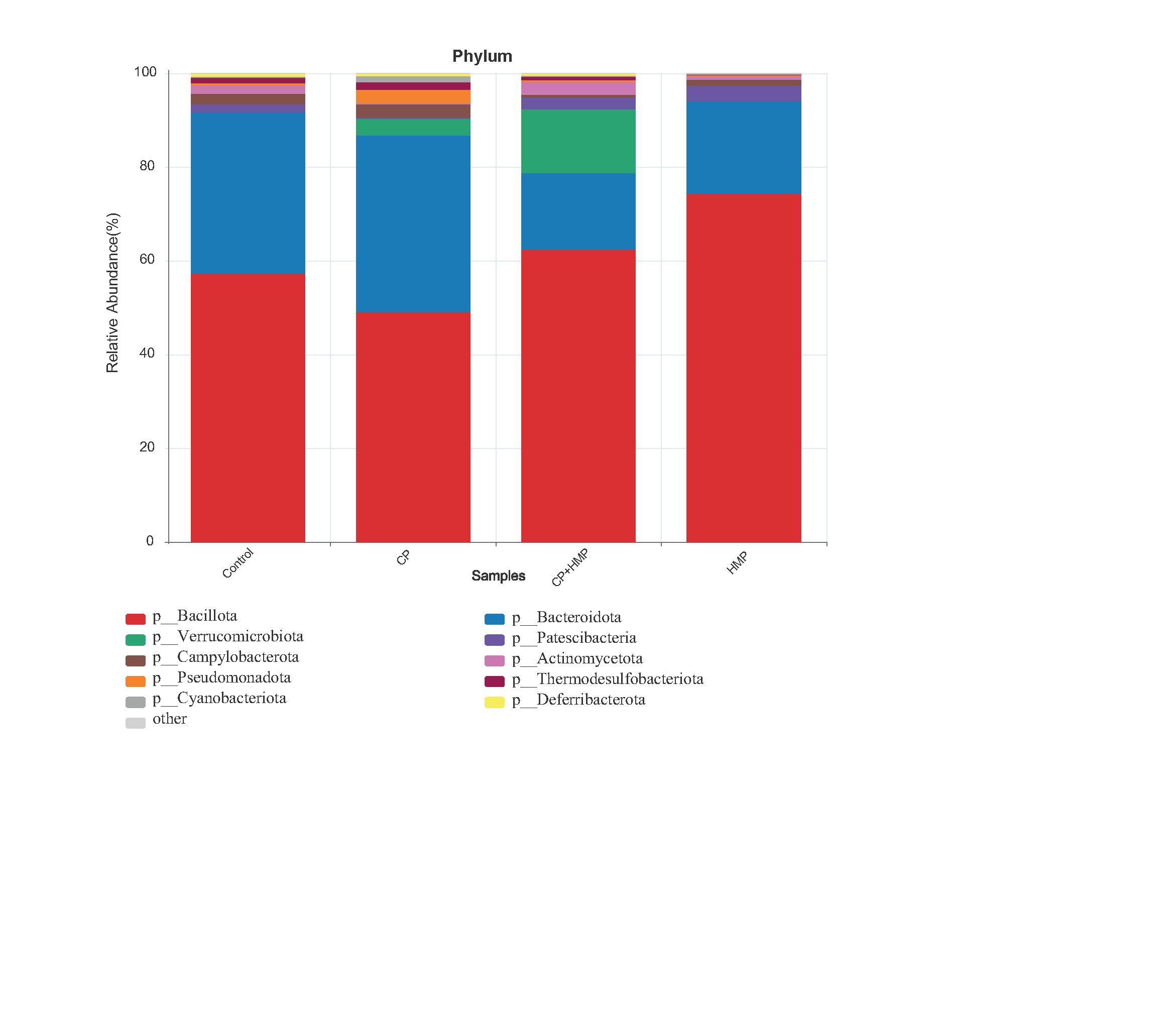


**Figure S1.** Relative abundance of gut microbiota at the phylum level among different experimental groups.

**
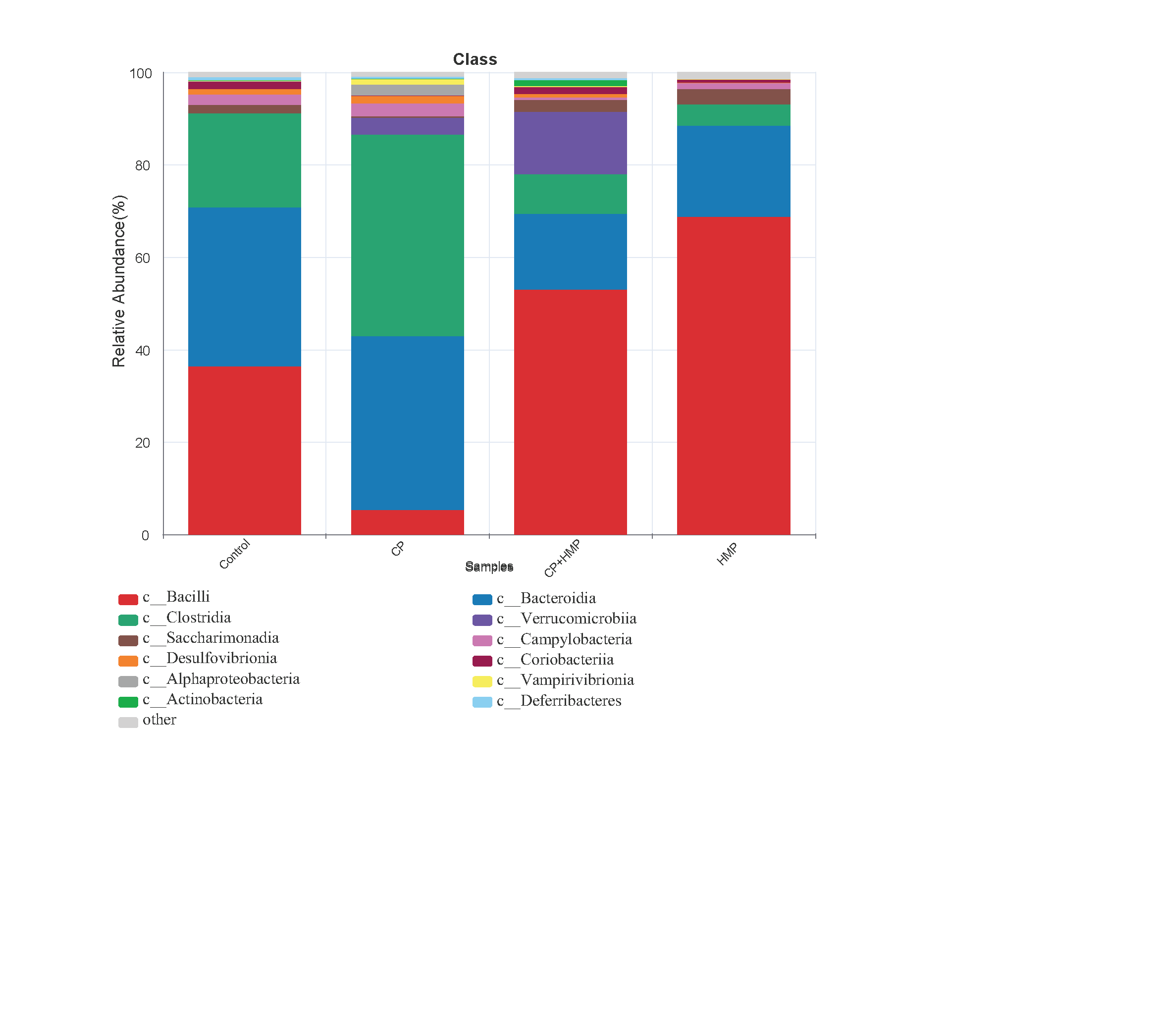
**

**Figure S2.** Relative abundance of gut microbiota at the class level among different experimental groups.


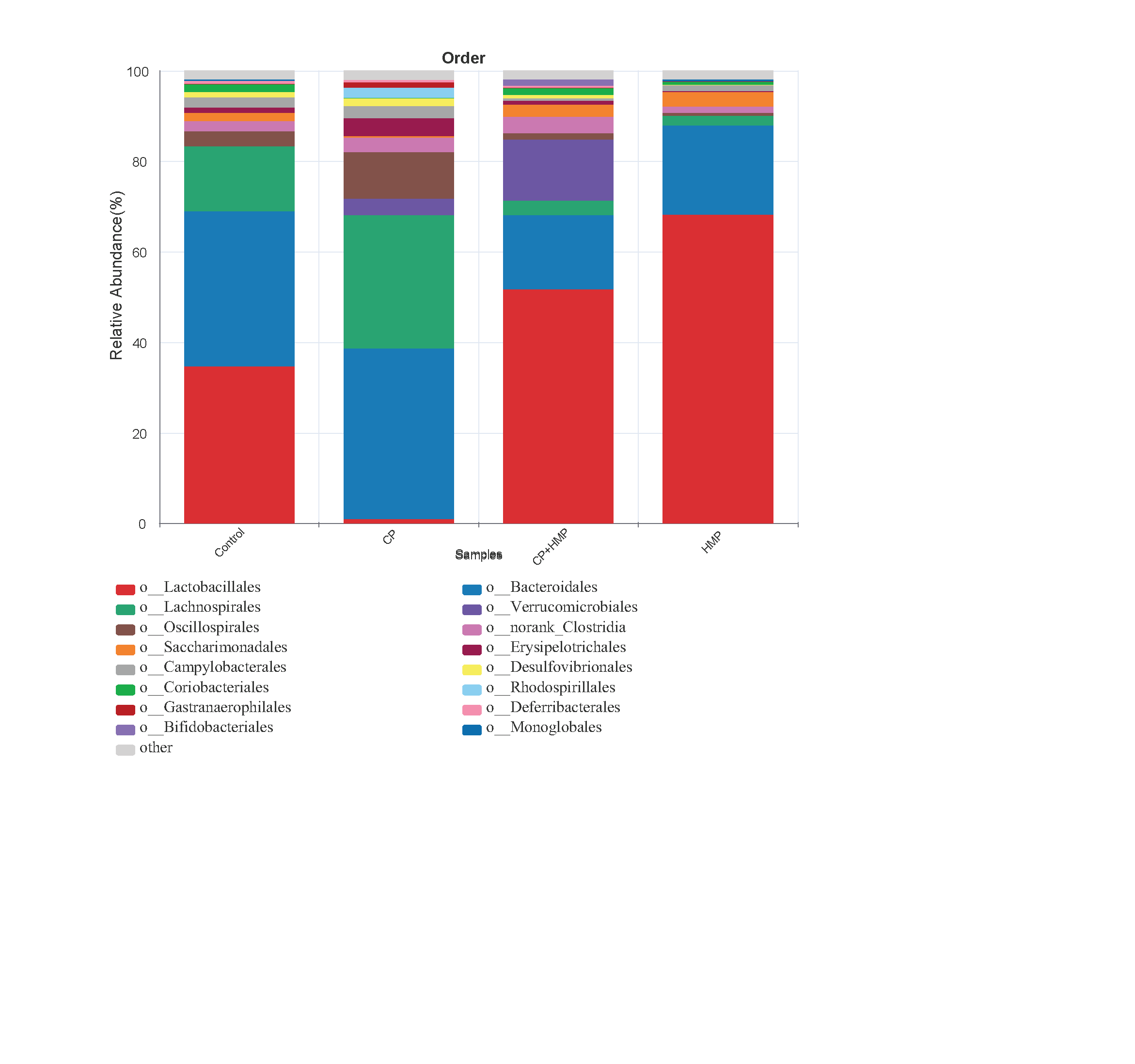


**Figure S3.** Relative abundance of gut microbiota at the order level among different experimental groups.


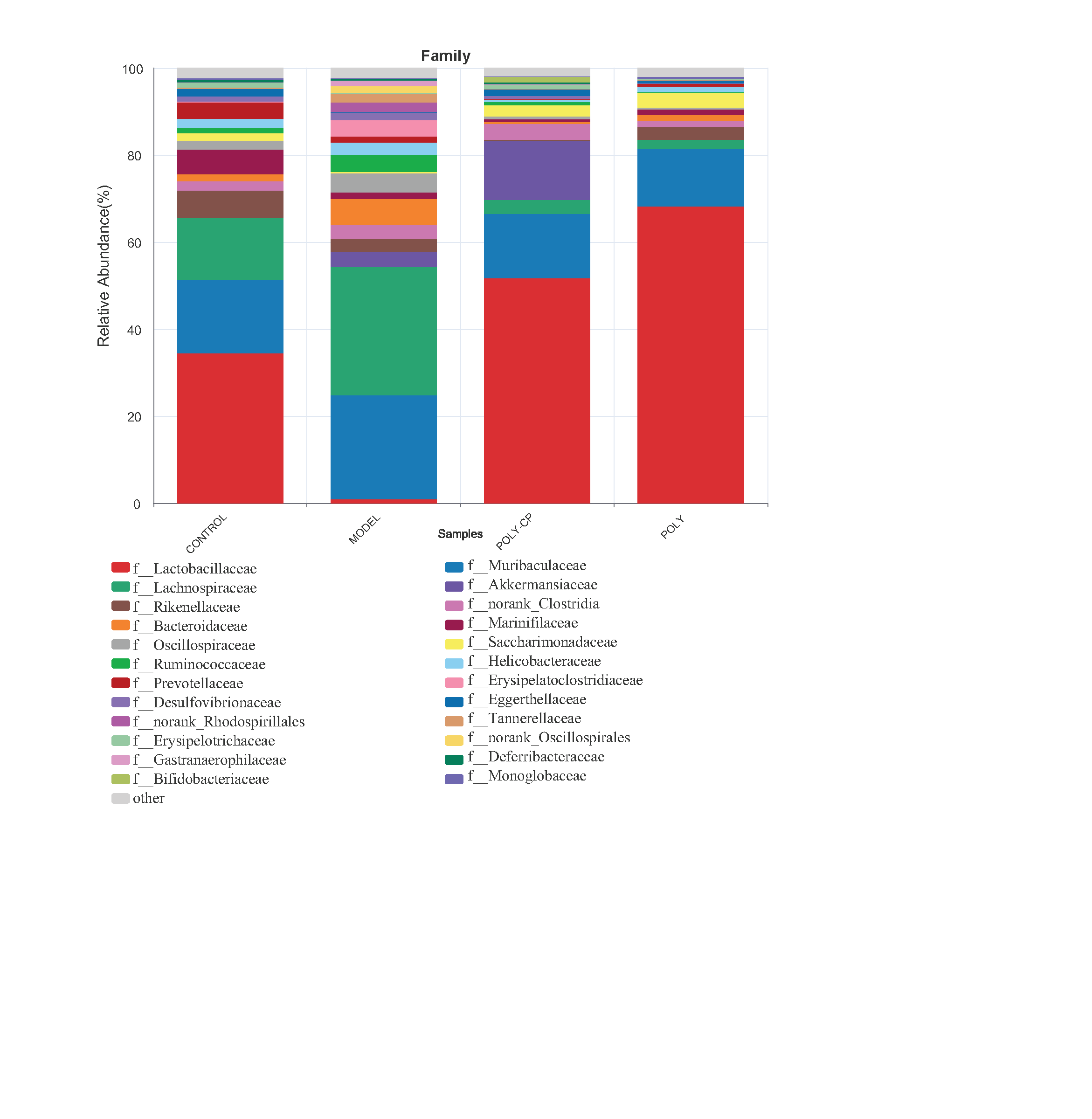


**Figure S4.** Relative abundance of gut microbiota at the family level among different experimental groups.


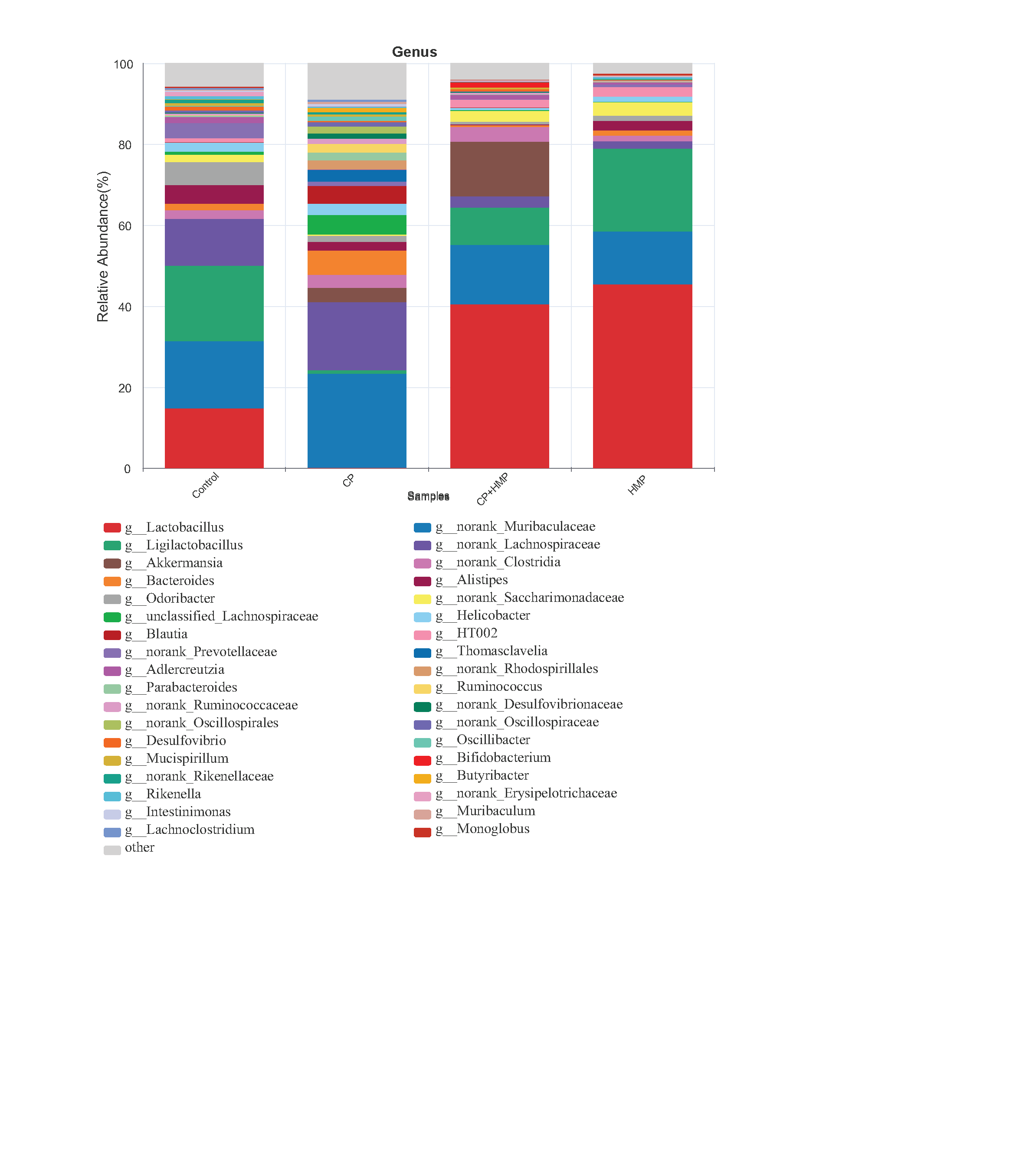


**Figure S5.** Relative abundance of gut microbiota at the genus level among different experimental groups.


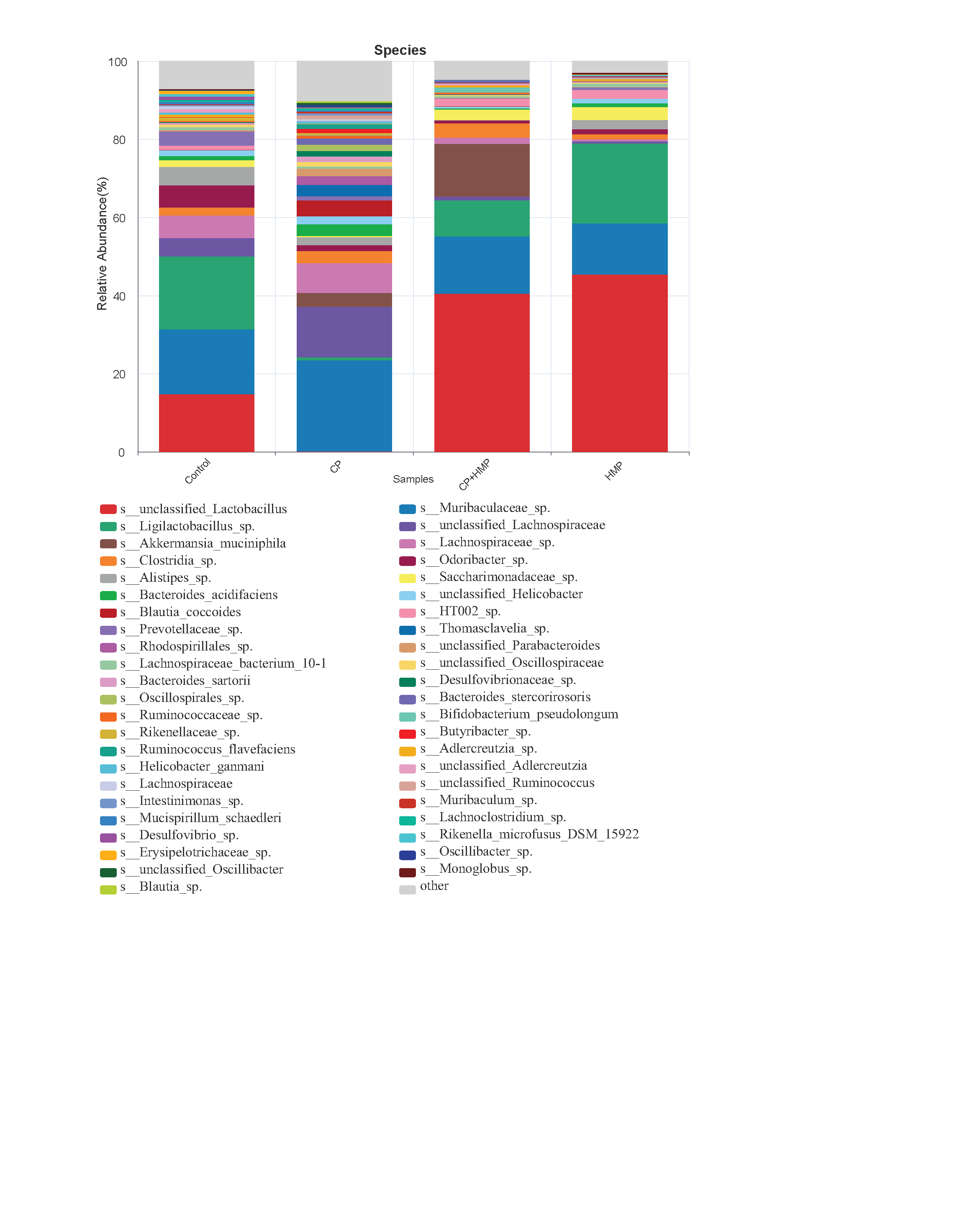


**Figure S6.** Relative abundance of gut microbiota at the species level among different experimental groups.


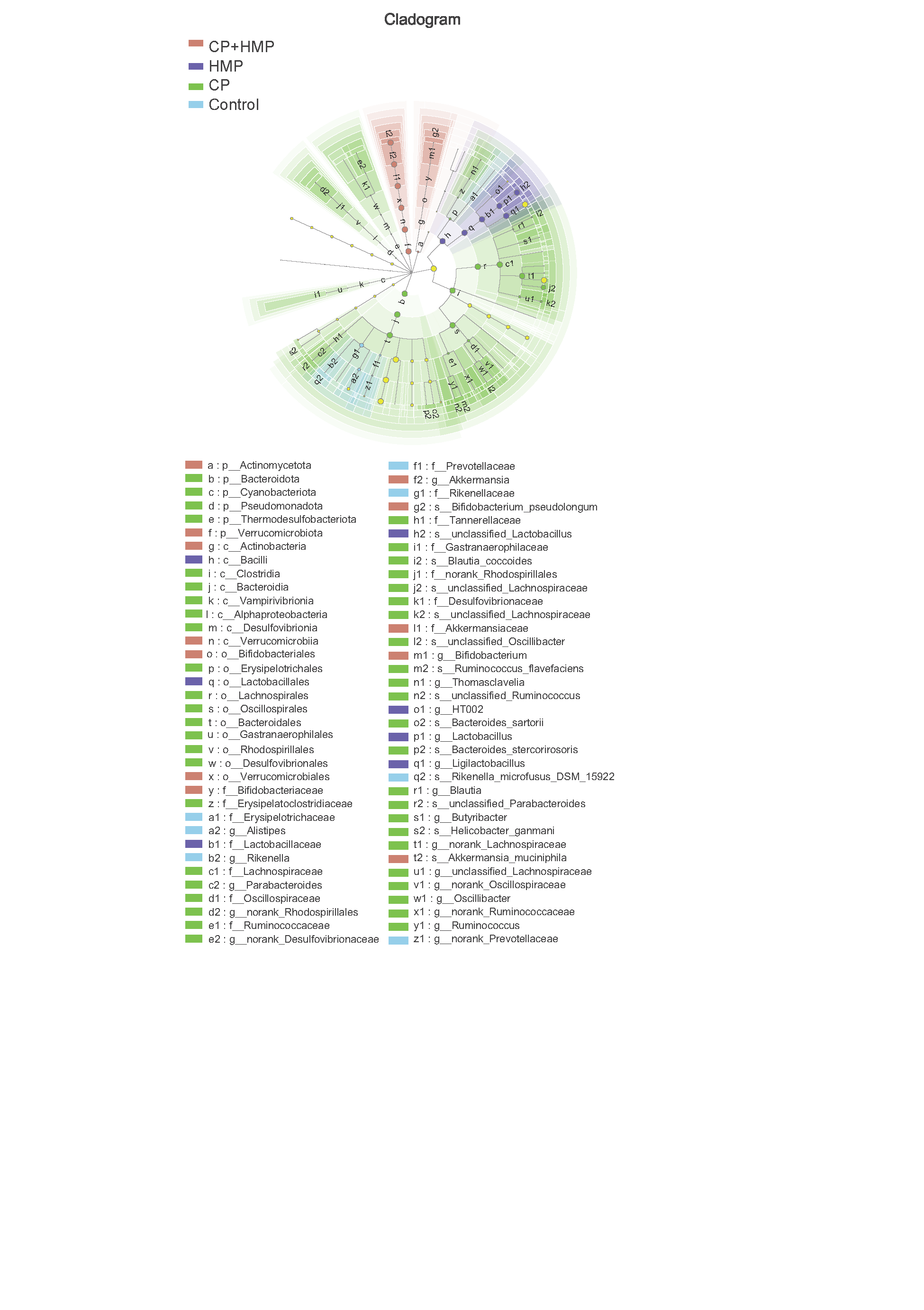


**Figure S7.** Cladogram depicting the taxonomic distribution of microbial biomarkers across experimental groups. The cladogram is based on Linear Discriminant Analysis (LDA) scores, with taxa color-coded by experimental treatment: Control (purple), HMP (green), CP (blue), and CP+HMP (red). The taxa at different taxonomic levels (phylum to genus) are represented in concentric circles, with nodes indicating group-specific biomarkers. The diagram highlights key shifts in microbial community composition.
